# Sensing and reversible capture of methylamine, methanethiol, and formic acid on Ti_2_C-based MXene monolayers: a DFT study

**DOI:** 10.1039/d6ra07364f

**Published:** 2026-11-02

**Authors:** Michael Qu, Xuan Luo

**Affiliations:** a National Graphene Research and Development Center USA qu.michael@outlook.com

## Abstract

Surface termination governs adsorption and electronic response in Ti_2_C MXene monolayers, but how O, F, and OH terminations shape sensing and reversible capture across distinct functional groups remains unresolved. Using dispersion-corrected first-principles DFT, we evaluated Ti_2_CO_2_, Ti_2_CF_2_, and Ti_2_C(OH)_2_ for the adsorption of methylamine (CH_3_NH_2_), methanethiol (CH_3_SH), and formic acid (HCOOH), selected to represent amine, thiol, and carboxyl groups, respectively. The adsorption energies ranged from −0.663 to −0.231 eV, and overall adsorption strength followed Ti_2_C(OH)_2_ > Ti_2_CO_2_ > Ti_2_CF_2_. Ti_2_CO_2_ and Ti_2_CF_2_ preferentially adsorbed CH_3_SH, whereas Ti_2_C(OH)_2_ preferred CH_3_NH_2_ and formed the strongest adsorption complex overall. Adsorption-energy-based molecular residence-time estimates ranged from 8.10 × 10^−8^ to 1.40 × 10^−1^ s, indicating preliminary desorption trends rather than established regeneration kinetics. Ti_2_CO_2_ remained semiconducting, with model-dependent band-gap-based electronic response estimates of 22.6–33.5% and work function shifts of −0.62 to +0.13 eV, supporting its potential for semiconductor sensing within the assumed conductivity model. Ti_2_C(OH)_2_ exhibited the strongest adsorption, particularly for CH_3_NH_2_; its subsecond residence-time estimates do not independently establish reversible capture performance. Its small, similar work function shifts of −0.05 to −0.03 eV indicated limited molecular differentiation through work function alone. Ti_2_CF_2_ exhibited the weakest adsorption and shortest residence-time estimates, while molecule-dependent work function shifts of −0.54 to +0.14 eV supported its potential for work-function-based sensing. These findings demonstrate that the strongest adsorption does not necessarily produce the most distinctive electronic response and establish distinct adsorption, preliminary desorption, and electronic-response profiles for the three Ti_2_C monolayers.

## Abstract 2

Surface termination tunes molecular adsorption and electronic responses in Ti_2_C MXenes, with the strongest adsorption not necessarily producing the most distinctive electronic response.

## Graphical abstract

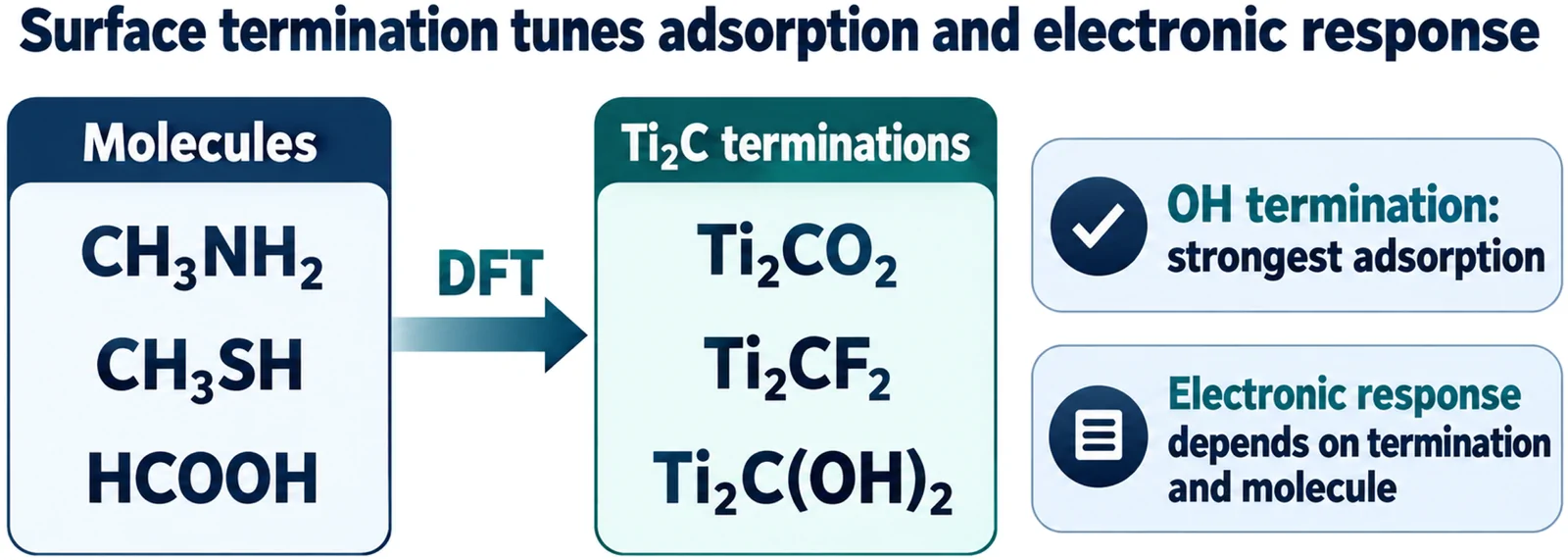

## 1. Introduction

Molecular recognition and capture at engineered interfaces are central to sensing and selective separation technologies.^1^ Adsorption-based sensing and reversible capture require a balance between retention and desorption: weak adsorbate–surface interactions can limit retention and produce minor electronic changes,^2,3^ whereas excessively strong interactions can hinder desorption and reduce reversibility.^4^ Molecular differentiation adds a further challenge because target molecules and interfering species may exhibit similar adsorption behavior or electronic responses in complex mixtures.^5,6^ These competing requirements motivate the development of reusable interfaces with tunable surface chemistries that balance sufficient molecular retention, reversible release, molecular differentiation, and measurable electronic responses rather than simply maximizing adsorption.

Two-dimensional materials are attractive for these applications,^7,8^ because they offer large accessible surface areas and can exhibit adsorption-induced electronic changes.^9,10^ Graphene, hexagonal boron nitride, transition metal dichalcogenides, and borocarbonitrides have been investigated for molecular sensing and capture.^11–14^ However, many pristine monolayers interact weakly with small neutral molecules, prompting defect engineering, doping, or covalent functionalization to strengthen adsorption and electronic response.^15–17^ Because these modifications can simultaneously alter local geometry, chemical composition, and electronic structure, attributing the resulting adsorption and electronic responses to a single factor can be difficult. Materials whose surface chemistry can be varied systematically while retaining the same underlying lattice therefore provide a more controlled basis for isolating the effects of surface chemistry on adsorption and electronic response.

MXenes are two-dimensional transition metal carbides, nitrides, and carbonitrides commonly synthesized by selectively removing the A-layer element from layered MAX phases, M_*n*+1_AX_*n*_.^18,19^ The resulting sheets generally contain surface terminations, denoted T_*x*_, and are described by the formula M_*n*+1_X_*n*_T_*x*_.^20^ Since Ti_3_C_2_T_*x*_ was first reported in 2011,^19,21^ MXenes have been investigated for energy storage,^22^ catalysis,^23^ separation,^24^ contaminant removal,^25^ and sensing.^26^ Their versatility arises from atomically accessible surfaces,^27,28^ tunable surface chemistry,^29^ and favorable electronic properties.^30^ O, OH, and F terminations introduced during etching and postsynthetic processing can create distinct local electronic environments,^29,31,32^ surface dipoles,^33^ and hydrogen-bonding characteristics.^34^ Naguib *et al.* reported OH and F terminations on the first Ti_3_C_2_ MXene,^19^ and subsequent first-principles studies showed that termination type and local termination arrangement can substantially alter MXene stability and electronic structure,^32,33,35,36^ establishing surface termination as a fundamental design variable.^37^ These termination-dependent changes can also influence adsorption strength and preferred adsorption geometries.^38,39^ Adsorption can further redistribute charge between the molecule and monolayer and modify surface dipoles,^33,34^ producing work function shifts that serve as surface-sensitive electronic signatures.^40^ This coupled tunability of adsorption and electronic response makes MXenes attractive materials for molecular sensing and reversible capture.^41–43^

As an M_2_X MXene, Ti_2_C provides a structurally simple platform for examining termination effects without the additional layer complexity of thicker titanium carbide MXenes.^37^ O-, F-, and OH-terminated Ti_2_C monolayers, denoted Ti_2_CO_2_, Ti_2_CF_2_, and Ti_2_C(OH)_2_, share the same Ti_2_C backbone while differing systematically in surface termination. Because experimentally synthesized Ti_2_CT_*x*_ commonly contains mixed O, F, and OH terminations,^44^ these uniformly terminated structures serve as well-defined model systems for evaluating the individual effects of O, F, and OH terminations. Previous first-principles studies have reported that Ti_2_CO_2_ is semiconducting,^32^ whereas Ti_2_CF_2_ and Ti_2_C(OH)_2_ are metallic.^35,36^ The distinct electronic properties of these monolayers determine how sensing response is evaluated: band gap changes serve as a metric for semiconducting Ti_2_CO_2_, whereas work function shifts and electronic structure changes near the Fermi level provide complementary indicators for all three monolayers.^33,40^

To systematically compare the adsorption of chemically and biologically relevant functional groups, we focused on amine, thiol, and carboxyl moieties. Amine groups are prevalent in amino acids, proteins,^45^ and other nitrogen-containing compounds; thiol groups occur in cysteine and related sulfur-containing biomolecules;^45^ and carboxyl groups are widespread in organic acids, amino acids, and oxidized organic matter.^46^ Accordingly, methylamine (CH_3_NH_2_), methanethiol (CH_3_SH), and formic acid (HCOOH) were selected as compact model adsorbates representing amine, thiol, and carboxyl groups, respectively. Their compact structures minimize conformational complexity and facilitate identification of functional-group-dependent adsorption trends.

Previous experimental and first-principles studies have demonstrated the potential of titanium carbide MXenes for molecular sensing and adsorptive capture.^47–50^ Kim *et al.* detected acetone and ethanol using metallic Ti_3_C_2_T_*x*_, demonstrating measurable adsorption-induced electrical responses in metallic MXene channels.^41^ For Ti_2_CO_2_, Yu *et al.* found that only NH_3_ chemisorbed among the small gas molecules examined, producing substantial charge transfer and pronounced changes in current–voltage behavior.^38^ Gouveia *et al.* reported preferential binding through N atoms for most of the examined amino acids on Ti_2_CO_2_.^39^ More directly, Thomas and Zaeem compared phosgene sensing on O-, F-, and OH-terminated Ti_2_C and found stable adsorption and charge transfer only on Ti_2_C(OH)_2_.^42^ Gouveia and Gomes evaluated 12 flue-gas molecules across nine Ti_2_C surface terminations and found termination-dependent adsorption behavior.^43^ Collectively, these studies indicate that molecular structure and surface termination jointly influence adsorption and electronic response.

However, systematic comparisons linking O-, F-, and OH-termination effects to functional-group-dependent adsorption, adsorbate preference, geometric sensitivity, recovery behavior, and complementary electronic responses across representative amine-, thiol-, and carboxyl-containing adsorbates remain limited. In particular, it remains unclear whether the termination that provides the strongest molecular retention also produces the most distinctive electronic response, or whether different terminations are better suited to semiconductor sensing, work-function-based sensing, or reversible capture. Resolving these questions is necessary to distinguish the potential roles of Ti_2_CO_2_, Ti_2_CF_2_, and Ti_2_C(OH)_2_ and identify the chemical factors underlying their different behavior.

In this study, we used dispersion-corrected first-principles DFT calculations to investigate the adsorption of CH_3_NH_2_, CH_3_SH, and HCOOH on Ti_2_CO_2_, Ti_2_CF_2_, and Ti_2_C(OH)_2_ monolayers and characterize the resulting electronic changes. Each molecule–monolayer pair was evaluated at three adsorption sites and in two orientations, yielding 54 initial configurations. Adsorption energies were used to identify the most stable adsorption complex for each molecule–monolayer pair. The resulting nine complexes were further analyzed using minimum adsorption distance and estimated molecular residence time to evaluate adsorbate–monolayer proximity and reversibility, along with charge density difference, band structure, and projected density of states (PDOS) analyses to examine charge redistribution and electronic changes. Adsorption-induced work function shifts were calculated to quantify molecule-dependent changes in surface potential and evaluate work-function-based sensing. For semiconducting Ti_2_CO_2_, adsorption-induced band gap changes were used to estimate model-dependent electronic responses. This systematic comparison reveals how O, F, and OH terminations affect adsorption strength, adsorbate preference, geometric sensitivity, reversibility, and electronic response, providing a comparative basis for further evaluation of Ti_2_C monolayers for molecular sensing or reversible capture.

## 2. Method

### 2.1 Computational details

All calculations were performed using density functional theory (DFT) as implemented in the ABINIT package.^51–53^ The exchange–correlation interactions were treated using the generalized gradient approximation (GGA) in the Perdew–Burke–Ernzerhof (PBE) form.^54^ Pseudopotentials were generated using the projector augmented-wave (PAW) method *via* the AtomPAW code,^55,56^ with the electron configurations and corresponding cutoff radii summarized in Table 1. The Grimme-D3 correction with Becke–Johnson damping was included to account for van der Waals interactions between molecules and MXene surfaces.^57,58^

**Table 1:** Electron configurations and radius cutoffs of elements used to generate PAW pseudopotentials

Element	Electron configuration	Radius cutoff (bohr)
Hydrogen (H)	1s^1^	0.99
Carbon (C)	[He]2s^2^2p^2^	1.51
Nitrogen (N)	[He]2s^2^2p^3^	1.20
Oxygen (O)	[He]2s^2^2p^4^	1.41
Fluorine (F)	[He]2s^2^2p^5^	1.40
Sulfur (S)	[Ne]3s^2^3p^4^	1.90
Titanium (Ti)	[Ar]3d^2^4 s^2^	2.30

The self-consistent-field (SCF) iterations were converged to a total-energy tolerance of 1.0 × 10^−8^ Ha. Convergence tests for the kinetic energy cutoff, Monkhorst–Pack *k*-point grids,^59^ and out-of-plane cell length were performed; they were considered converged when the total energy variation between two consecutive data sets fell below 0.0001 Ha. The converged kinetic-energy cutoffs, PAW double-grid cutoffs, and *k*-point meshes varied according to the system and calculation type; the calculation-specific values are provided in the complete ABINIT input files deposited in the Zenodo repository cited in the Data availability section. A Fermi–Dirac occupation smearing of 0.002 Ha was applied,^60^ and a total cell height of approximately 20 Å was used in the non-periodic direction for production relaxation; the vacuum separation is smaller by the full atomic *z* extent. A concise parameter table and coordinate-derived vacuum gaps are provided in SI Section S1; calculation-specific work-function cell heights and extraction diagnostics are given in SI Section S5.

Structural optimizations employed the Broyden–Fletcher–Goldfarb–Shanno (BFGS) algorithm,^61^ with a force tolerance of 5.0 × 10^−4^ Ha bohr^−1^ and a total-energy tolerance of 1.0 × 10^−8^ Ha. Convergence exceptions in the additional tests are identified in SI Sections S2–S4.

### 2.2 Atomic structures

Because many target species in sensing applications interact with surfaces through recurring functional groups, three small molecules were selected as compact molecular proxies: methylamine (CH_3_NH_2_) for amine groups, methanethiol (CH_3_SH) for thiol groups, and formic acid (HCOOH) for carboxyl groups. The isolated-molecule structures (Fig. 1) use cubic cell lengths of 20, 16, and 15 Å for CH_3_NH_2_, CH_3_SH, and HCOOH, respectively; calculation-specific settings are retained in the archived inputs. The substrates were O-, F-, and OH-terminated Ti_2_C MXene monolayers, denoted Ti_2_CO_2_, Ti_2_CF_2_, and Ti_2_C(OH)_2_.

**Fig. 1:**
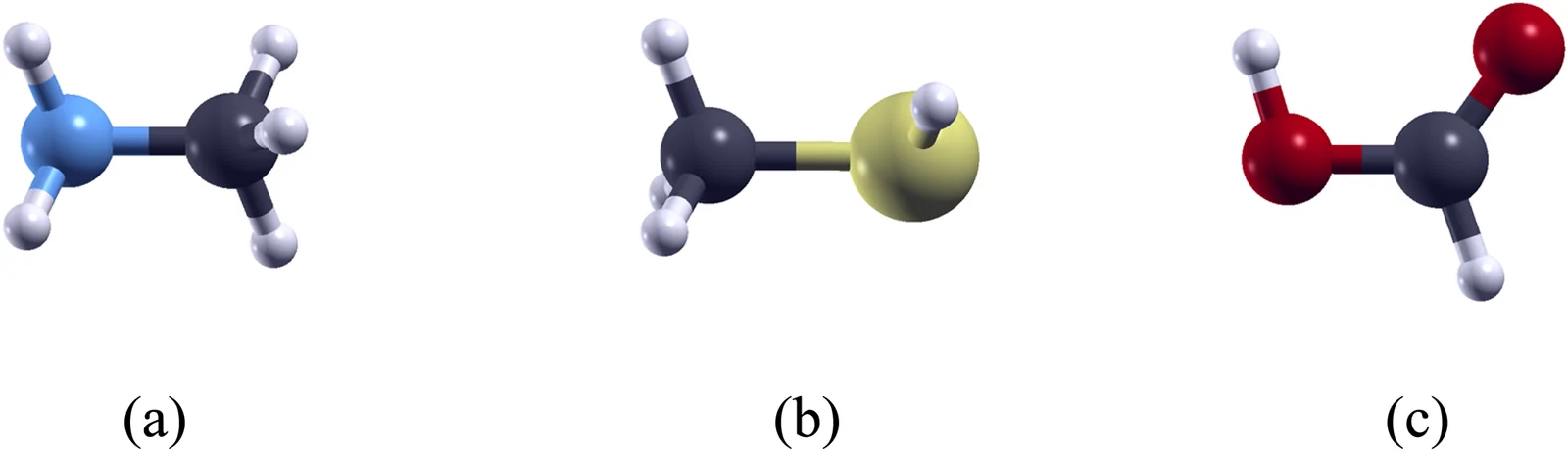
Atomic structures of (a) methylamine (CH_3_NH_2_), (b) methanethiol (CH_3_SH), and (c) formic acid (HCOOH). H, C, O, N, and S atoms are shown in white, black, red, blue, and yellow, respectively.

For each molecule–monolayer pair, three high-symmetry adsorption sites were evaluated: a top site (T) directly above a termination atom, a bridge site (B) above a Ti–termination bond, and a hollow site (H) at the center of the surface hexagonal lattice. At each site, two molecular orientations were examined: parallel (P), with the molecular backbone approximately parallel to the surface, and vertical (V), with the molecular backbone approximately perpendicular to the surface. This yields six configurations per molecule–monolayer pair (T/P, T/V, B/P, B/V, H/P, and H/V), as illustrated in Fig. 2 for Ti_2_CO_2_, and 54 configurations in total across the three molecules and three monolayers.

**Fig. 2:**
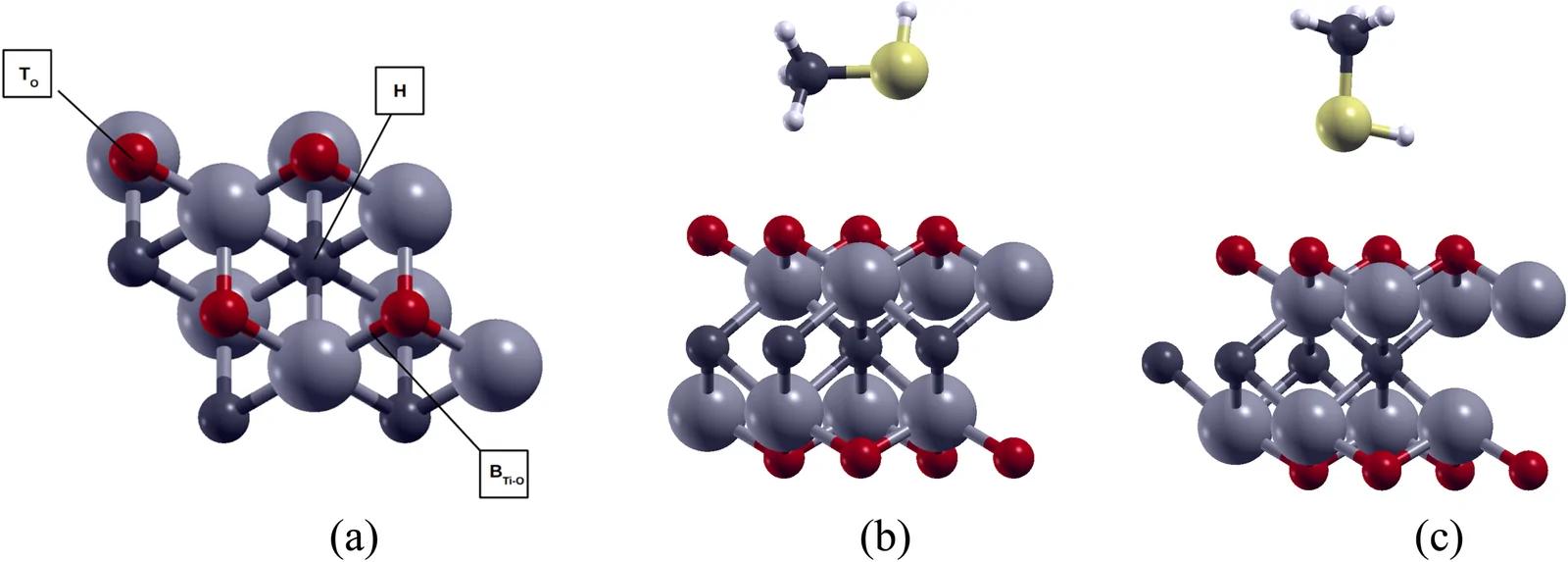
(a) Top view of the Ti_2_CO_2_ monolayer showing the representative adsorption sites: the top site above a surface O atom (T_O_), the bridge site above a Ti–O bond (B_Ti–O_), and the hollow site at the center of the surface lattice (H). Representative CH_3_SH/Ti_2_CO_2_ configurations in the (b) parallel and (c) vertical orientations. H, C, S, O, and Ti atoms are shown in white, black, yellow, red, and gray, respectively.

For each termination, the primitive (1 × 1) cell was optimized by relaxing the in-plane lattice parameters while preserving hexagonal symmetry, yielding the reference lattice constants in Table 3. The production 2 × 2 structures instead use a common in-plane cell length of 6.05998 Å (primitive *a* ≈ 3.02999 Å), as shown in Fig. 3(a), with a total out-of-plane cell height of approximately 20 Å. Relative to the Table 3 reference constants, the common cell imposes strains of approximately +0.60%, −0.10%, and −0.49% for O, F, and OH termination. The effect of using the separately optimized lattice constants instead of the common lattice constant was assessed for three representative adsorption complexes (SI Section S2).

**Fig. 3:**
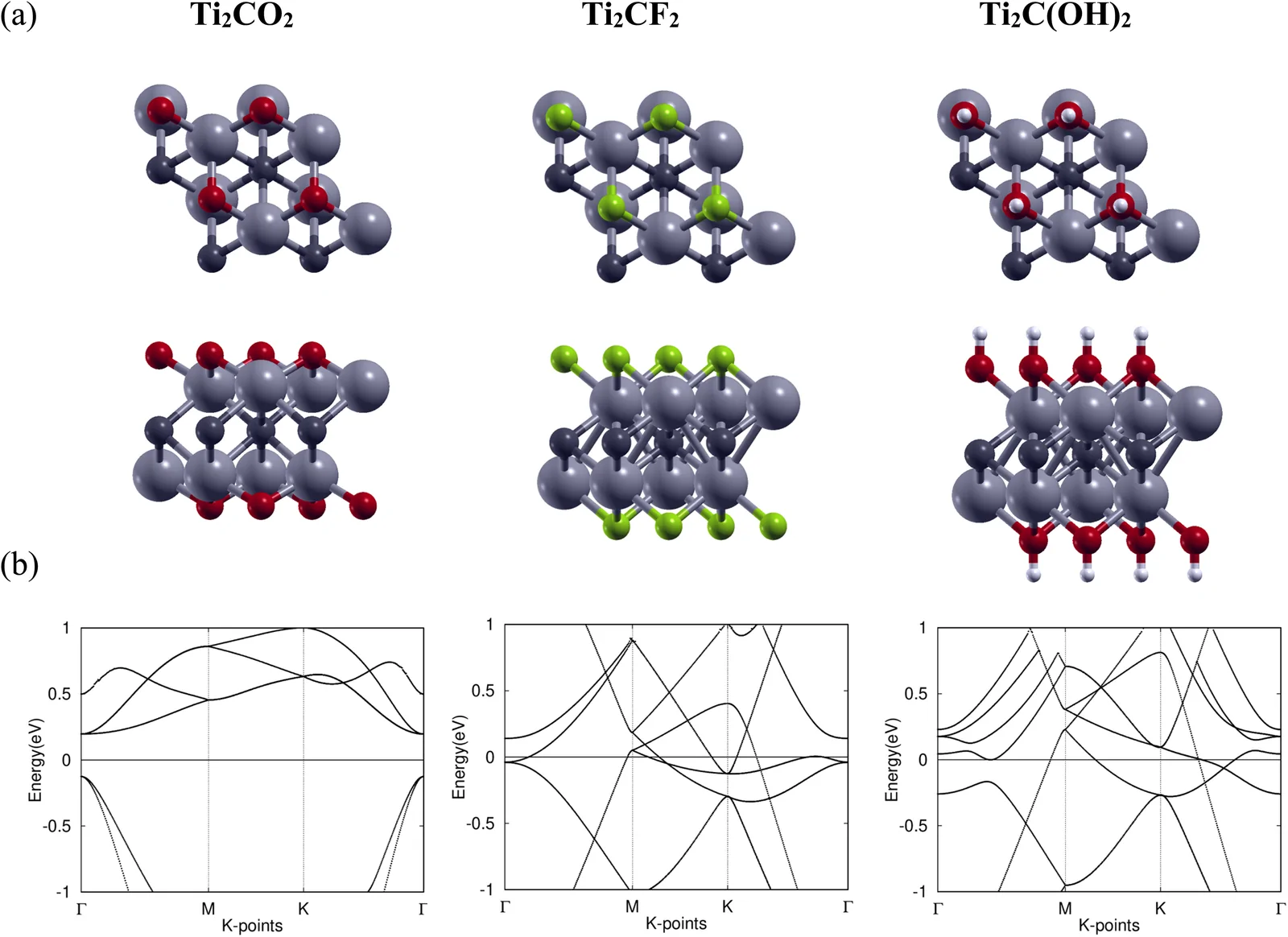
(a) Top and side views of the Ti_2_CO_2_, Ti_2_CF_2_, and Ti_2_C(OH)_2_ monolayers. H, C, O, F, and Ti atoms are shown in white, black, red, green, and gray, respectively. (b) Electronic band structures of Ti_2_CO_2_, Ti_2_CF_2_, and Ti_2_C(OH)_2_ monolayers, with the Fermi level set to 0 eV.

The 54 initial configurations were relaxed, and the lowest-energy converged configuration examined for each molecule–monolayer pair was retained for detailed analysis. The original vertical CH_3_NH_2_ starts are methyl-down; nine additional nitrogen-facing starts and convergence flags are reported in SI Table S3. The F hollow/parallel and OH bridge/N-down CH_3_NH_2_ runs did not meet the production force target and are excluded from definitive minimum selection. The configuration search is not exhaustive.

### 2.3 Energy calculations

Total energies were evaluated for the isolated molecules, the corresponding bare O-, F-, and OH-terminated Ti_2_C monolayers, and the relaxed molecule–monolayer adsorption complexes. The adsorption energy, *E*_ad_, was calculated as

1 *E*_ad_ = *E*_complex_ − *E*_monolayer_ − *E*_mol_

where *E*_complex_, *E*_monolayer_, and *E*_mol_ are the total energies of the relaxed molecule–monolayer complex, the corresponding bare monolayer, and the isolated molecule, respectively. The minimum adsorption distance, *d*_min_, was defined as the shortest distance between any atom of the adsorbed molecule and any atom of the monolayer after structural relaxation.

To assess supercell-size effects, 3 × 3 supercell calculations were performed for the nine most stable adsorption complexes identified in the 2 × 2 screening, using the same adsorption configuration and convergence criteria. The absolute adsorption-energy difference was calculated as

2 |Δ*E*| = |*E*_ad,3×3_ − *E*_ad,2×2_|

where *E*_ad,3×3_ and *E*_ad,2×2_ are the adsorption energies obtained from the 3 × 3 and 2 × 2 supercell calculations, respectively.

### 2.4 Electronic band structure

Band structures were computed for the bare monolayers and the most stable adsorption complex of each molecule–monolayer pair along the *Γ*–*M*–*K*–*Γ* path of the hexagonal Brillouin zone, with the Fermi level set to zero. The reported band gaps are PBE Kohn–Sham values.

### 2.5 Projected density of states

The projected density of states (PDOS) was computed using the tetrahedron method.^62^ The atoms nearest to the adsorption region were selected for projection. The PDOS of the 3d orbital of Ti, the 2p orbital of the surface termination atom (O or F), the 2p orbital of N in CH_3_NH_2_, the 3p orbital of S in CH_3_SH, the 2p orbital of O in HCOOH, and the 2p orbital of C and 1s orbital of H were plotted for the adsorption complexes.

### 2.6 Work function

To complement the band-structure analysis with a quantity applicable to both semiconducting and metallic monolayers, the work function was calculated as

3 *Φ* = *E*_vac_ − *E*_F_

where *E*_F_ is the Fermi energy and *E*_vac_ is the mean planar-averaged electrostatic potential in a 4 Å-wide periodic window centered half a cell away from the mean atomic *z* coordinate. No dipole correction was applied. This geometric window does not establish a flat adsorbate-side vacuum plateau; residual variation and bare/complex cell-height differences are documented in SI Section S5. The adsorption-induced shift, Δ*Φ* = *Φ*_complex_ − *Φ*_bare_, is used as a complementary electronic response indicator, particularly for metallic Ti_2_CF_2_ and Ti_2_C(OH)_2_. It combines vacuum-level and Fermi-level changes, so it is not an independent measure of the interfacial dipole or electron-transfer direction.^33,40^

### 2.7 Sensing response

For systems retaining a finite band gap, the sensing response was estimated from the corresponding conductivity change.^63–65^ The percentage band-gap change was calculated as4
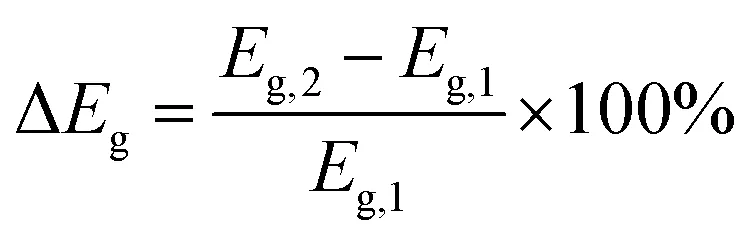
where *E*_g,1_ and *E*_g,2_ are the band gaps before and after adsorption. The electrical conductivity was estimated *via*5
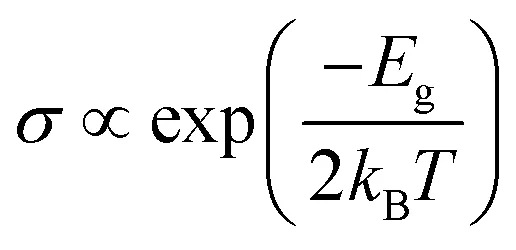
where *E*_g_ is the band gap, *k*_B_ is the Boltzmann constant, and *T* is the temperature. The sensing response was then calculated from the relative change in estimated conductivity before and after adsorption6
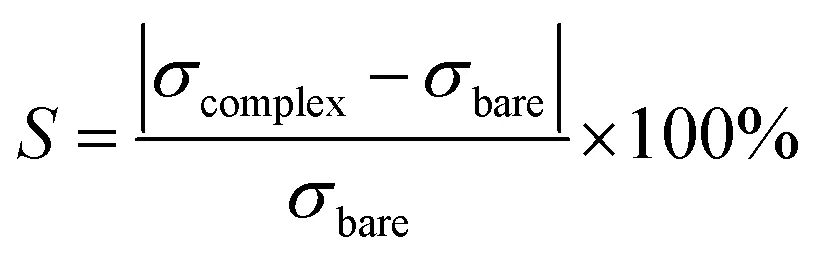
where *σ*_bare_ and *σ*_complex_ are the estimated conductivities of the bare monolayer and adsorption complex. For metallic terminations, Δ*Φ* serves as the primary electronic response indicator. Eqn (5) and (6) assume an intrinsic semiconductor with an unchanged conductivity prefactor and omit changes in mobility, doping and contacts. The percentages are therefore model-dependent electronic response estimates at the studied coverage, not device sensitivities.

### 2.8 Molecular residence time

The molecular residence time (*τ*), used as a preliminary desorption indicator, was estimated using the transition-state-theory approximation:^66^7
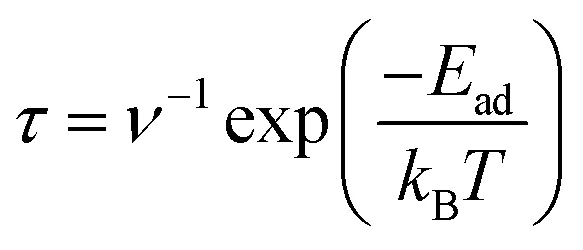
where *ν* = 10^12^ s^−1^ is the attempt frequency, *E*_ad_ is the adsorption energy, *k*_B_ = 8.617 × 10^−5^ is the Boltzmann constant, and *T* = 300 K is the temperature. Here |*E*_ad_| approximates the desorption barrier, and *k*_B_ has units of eV K^−1^. This molecular estimate is distinct from macroscopic sensor recovery; sensitivity to the assumed barrier, attempt frequency and temperature is reported in SI Section S6.

### 2.9 Charge density difference

The charge-density difference was computed as

8 Δ*ρ* = *ρ*_complex_ − *ρ*_monolayer_ − *ρ*_mol_

where *ρ*_complex_, *ρ*_monolayer_, and *ρ*_mol_ are the charge densities of the adsorption complex, the bare monolayer, and the isolated molecule, respectively, each evaluated at the geometry within the relaxed adsorption complex on a common real-space grid. Quantitative redistribution profiles derived from density data exported from ABINIT PAW calculations are reported in SI Section S7 and interpreted as representation-dependent descriptors.

## 3. Results and discussion

We first validated the structures of CH_3_NH_2_, CH_3_SH, and HCOOH and established structural and electronic baselines for the bare Ti_2_CO_2_, Ti_2_CF_2_, and Ti_2_C(OH)_2_ monolayers. The 54 initial adsorption configurations were then compared to determine the effects of surface termination, adsorption site, and molecular orientation on adsorption strength and adsorbate preference. The nine most stable complexes were subsequently characterized using adsorption energy, minimum adsorption distance, and estimated molecular residence time to compare molecular retention, adsorbate–monolayer proximity, and reversibility. Charge density difference, band structure, and PDOS analyses were used to examine charge redistribution and electronic structure changes. Work function shifts were calculated for all three monolayers to assess work-function-based sensing potential, whereas band-gap-based model-dependent electronic responses were estimated for semiconducting Ti_2_CO_2_.

### 3.1 Structural validation and electronic baselines

To establish a controlled baseline for evaluating adsorption-induced changes, we first optimized the isolated molecules and the bare Ti_2_CO_2_, Ti_2_CF_2_, and Ti_2_C(OH)_2_ monolayers. The molecular structures of CH_3_NH_2_, CH_3_SH, and HCOOH are shown in Fig. 1, and their calculated structural parameters and corresponding previously reported values are summarized in Table 2.^67–71^ For CH_3_NH_2_, the calculated C–N and N–H bond lengths are 1.465 and 1.021 Å, respectively, with H–N–H and H–N–C bond angles of 106.3 and 110.5°. For CH_3_SH, the C–S and S–H bond lengths are 1.820 and 1.350 Å, respectively, and the H–S–C bond angle is 97.0°. HCOOH exhibits C=O, C–O, and O–H bond lengths of 1.208, 1.352, and 0.982 Å, respectively, with O–C–O and H–O–C bond angles of 125.3 and 106.9°. Relative to experimental values,^68,70^ the deviations are 0.1–1.6% for bond lengths and 0.3–3.3% for bond angles. The largest deviation of 3.3% occurs for the H–S–C angle of CH_3_SH; however, the calculated value of 97.0° closely matches the reported theoretical value of 97.3°,^69^ differing by only 0.3°. These close agreements with the previously reported structural values validate the optimized molecular geometries used in subsequent analyses.

**Table 2:** Comparison of the calculated, previous theoretical, and experimental structural parameters of CH_3_NH_2_, CH_3_SH, and HCOOH, including bond lengths, bond angles, and percentage errors relative to experimental values^a^

System	Parameter	Calculated	Previous theoretical	Experimental	Error (%)
CH_3_NH_2_	C–N (Å)	1.465	1.47 (ref. 67)	1.471 (ref. 68)	0.4
	N–H (Å)	1.021	1.01 (ref. 67)	1.018 (ref. 68)	0.3
	C–H (Å)	1.101	1.09 (ref. 67)	1.093 (ref. 68)	0.7
	∠H–N–H (°)	106.3	106.0 (ref. 67)	105.8 (ref. 68)	0.5
	∠H–N–C (°)	110.5	111.0 (ref. 67)	111.0 (ref. 68)	0.5
CH_3_SH	C–S (Å)	1.820	1.83 (ref. 69)	1.818 (ref. 70)	0.1
	S–H (Å)	1.350	1.34 (ref. 69)	1.329 (ref. 70)	1.6
	C–H (Å)	1.096	1.09 (ref. 69)	1.104 (ref. 70)	0.7
	∠H–S–C (°)	97.0	97.3 (ref. 69)	100.3 (ref. 70)	3.3
	∠H–C–H (°)	109.2	108.8, 110.4 (ref. 69)	110.3 (ref. 70)	1.0
HCOOH	C=O (Å)	1.208	1.21 (ref. 71)	1.202 (ref. 70)	0.5
	C–O (Å)	1.352	1.35 (ref. 71)	1.343 (ref. 70)	0.7
	C–H (Å)	1.106	1.102 (ref. 71)	1.097 (ref. 70)	0.8
	O–H (Å)	0.982	0.977 (ref. 71)	0.972 (ref. 70)	1.0
	∠O–C–O (°)	125.3	125.1 (ref. 71)	124.9 (ref. 70)	0.3
	∠H–C–O (°)	125.4	125.0 (ref. 71)	124.1 (ref. 70)	1.0
	∠H–O–C (°)	106.9	107.4 (ref. 71)	106.3 (ref. 70)	0.6

a Two inequivalent H–C–H angles of 108.8° and 110.4° were reported.

The atomic structures and electronic band structures of Ti_2_CO_2_, Ti_2_CF_2_, and Ti_2_C(OH)_2_ monolayers are presented in Fig. 3, and their structural and electronic properties are summarized in Table 3. The calculated lattice constants are 3.012 Å for Ti_2_CO_2_, 3.033 Å for Ti_2_CF_2_, and 3.045 Å for Ti_2_C(OH)_2_, which closely agree with reported theoretical values,^72^ differing by only 0.1–0.2%. As shown in Fig. 3(b) and Table 3, Ti_2_CO_2_ has a calculated band gap of 0.322 eV, indicating semiconducting character,^32,73^ whereas bands cross the Fermi level for Ti_2_CF_2_ and Ti_2_C(OH)_2_, consistent with metallic character.^35,36^ These results validate the calculated structural parameters and electronic properties of the three Ti_2_C monolayers and establish distinct electronic baselines for evaluating adsorption-induced changes.

**Table 3:** Optimized structural and electronic properties of Ti_2_CO_2_, Ti_2_CF_2_, and Ti_2_C(OH)_2_ monolayers, including calculated and previous theoretical lattice constant (*a*), monolayer thickness (*t*), representative Ti–C bond length (*d*_Ti–C_), Ti–terminal atom bond length (*d*_Ti–T_), O–H bond length (*d*_O–H_), and band gap (*E*_g_)^a^

System	*a*, calculated (Å)	*a*, previous theoretical (Å)	*t* (Å)	*d*_Ti–C_ (Å)	*d*_Ti–T_ (Å)	*d*_O–H_ (Å)	*E*_g_ (eV)
Ti_2_CO_2_	3.012	3.018 (ref. 72)	4.41	2.180	1.970 (Ti–O)	N/A	0.322
Ti_2_CF_2_	3.033	3.036 (ref. 72)	4.80	2.088	2.153 (Ti–F)	N/A	0.000
Ti_2_C(OH)_2_	3.045	3.052 (ref. 72)	6.79	2.097	2.159 (Ti–O)	0.974	0.000

a T denotes the terminal atom bonded to Ti: O in Ti_2_CO_2_, F in Ti_2_CF_2_, and O in Ti_2_C(OH)_2_. The lattice constants are separately optimized references, whereas the thicknesses are geometric *z* extents of the supplied common-cell production structures (*a* ≈ 3.02999 Å); see SI Section S1.

### 3.2 Adsorption strength and adsorbate preference

To determine how surface termination, adsorption site, and molecular orientation influence adsorption strength and adsorbate preference, we examined 54 initial adsorption configurations comprising three molecules, CH_3_NH_2_, CH_3_SH, and HCOOH; three monolayers, Ti_2_CO_2_, Ti_2_CF_2_, and Ti_2_C(OH)_2_; three adsorption sites, top, bridge, and hollow; and two molecular orientations, parallel and vertical. These combinations yielded six configurations per molecule–monolayer pair and 18 configurations per monolayer. Fig. 4–6 show the optimized adsorption configurations, and Fig. 7 summarizes the corresponding adsorption energies. Applying the same adsorption sites and molecular orientations to every molecule–monolayer pair enables a controlled comparison of the effects of surface termination and adsorption geometry. The extended CH_3_NH_2_ orientation results and convergence flags are provided separately in SI Table S3.

**Fig. 4:**
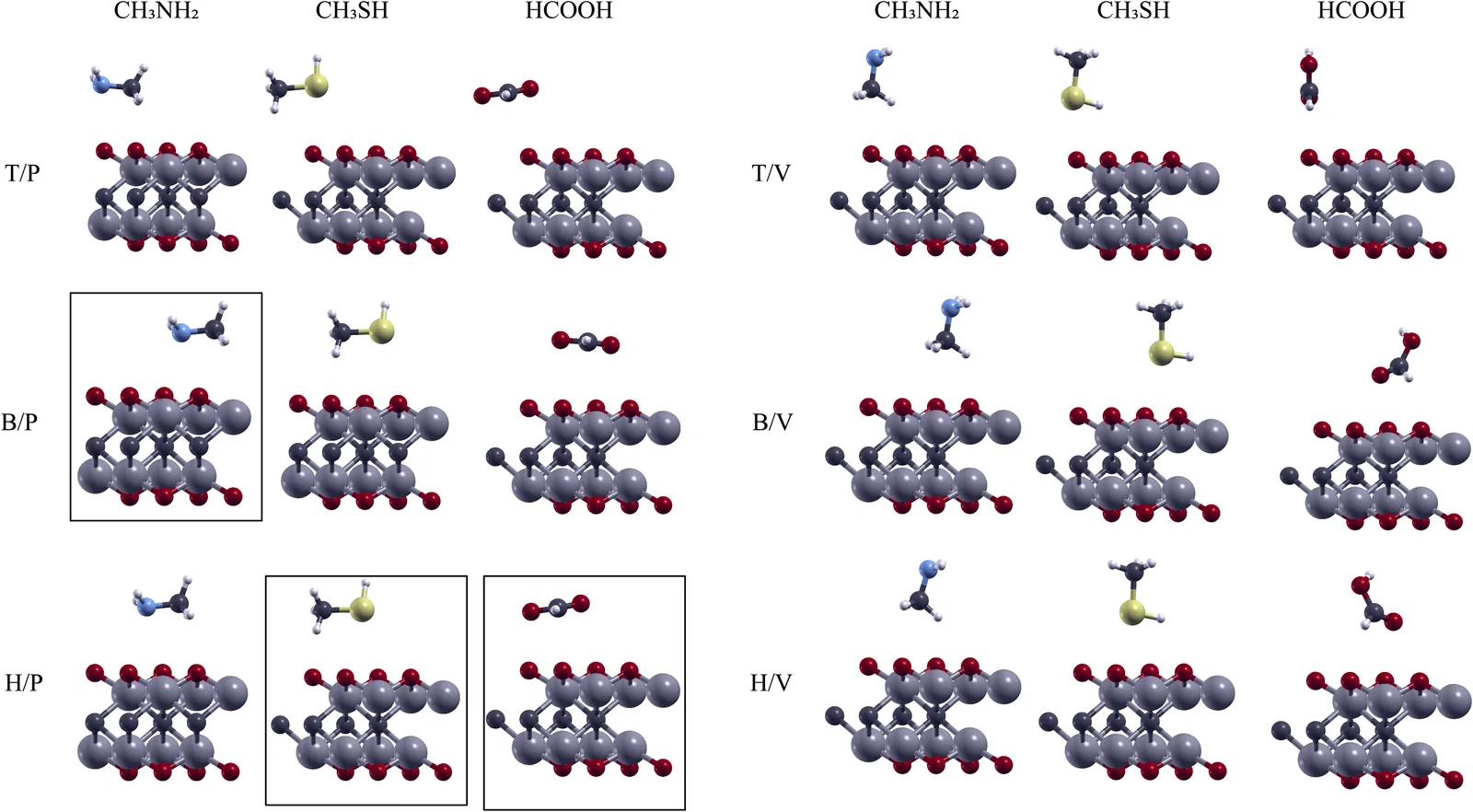
Side views of the optimized adsorption configurations of CH_3_NH_2_, CH_3_SH, and HCOOH on the Ti_2_CO_2_ monolayer. Configurations are labeled T/P, T/V, B/P, B/V, H/P, and H/V, where T, B, and H denote top, bridge, and hollow sites, and P and V denote parallel and vertical orientations, respectively. Black boxes indicate the most stable configuration for each molecule. H, C, N, O, S, and Ti atoms are shown in white, black, blue, red, yellow, and gray, respectively.

**Fig. 5:**
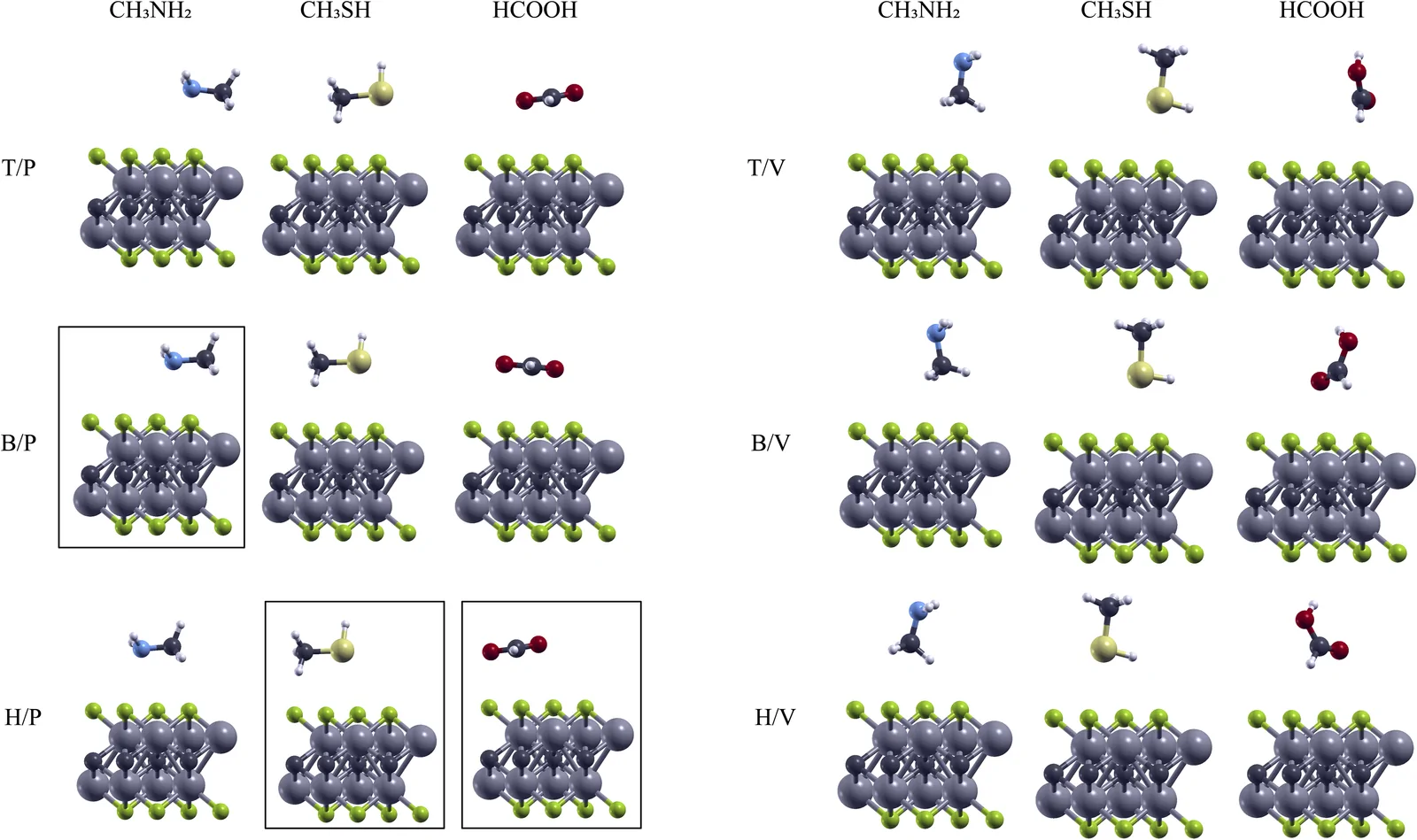
Side views of the optimized adsorption configurations of CH_3_NH_2_, CH_3_SH, and HCOOH on the Ti_2_CF_2_ monolayer. Configurations are labeled T/P, T/V, B/P, B/V, H/P, and H/V, where T, B, and H denote top, bridge, and hollow sites, and P and V denote parallel and vertical orientations, respectively. Black boxes indicate the most stable configuration for each molecule. H, C, N, O, S, F, and Ti atoms are shown in white, black, blue, red, yellow, green, and gray, respectively. The F hollow/parallel CH_3_NH_2_ run did not reach the force target and is shown for transparency, not as a converged minimum.

**Fig. 6:**
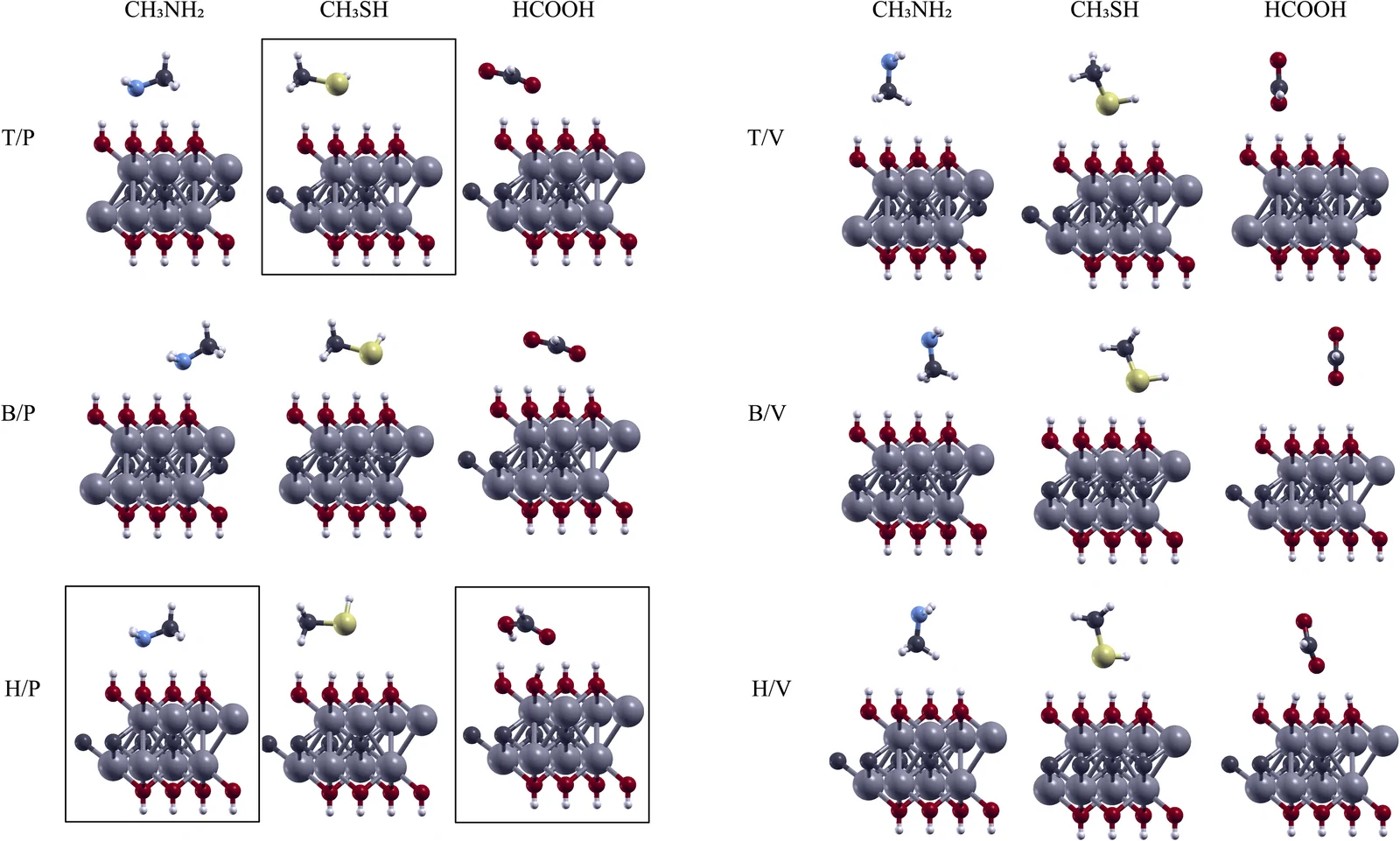
Side views of the optimized adsorption configurations of CH_3_NH_2_, CH_3_SH, and HCOOH on the Ti_2_C(OH)_2_ monolayer. Configurations are labeled T/P, T/V, B/P, B/V, H/P, and H/V, where T, B, and H denote top, bridge, and hollow sites, and P and V denote parallel and vertical orientations, respectively. Black boxes indicate the most stable configuration for each molecule. H, C, N, O, S, and Ti atoms are shown in white, black, blue, red, yellow, and gray, respectively.

**Fig. 7:**
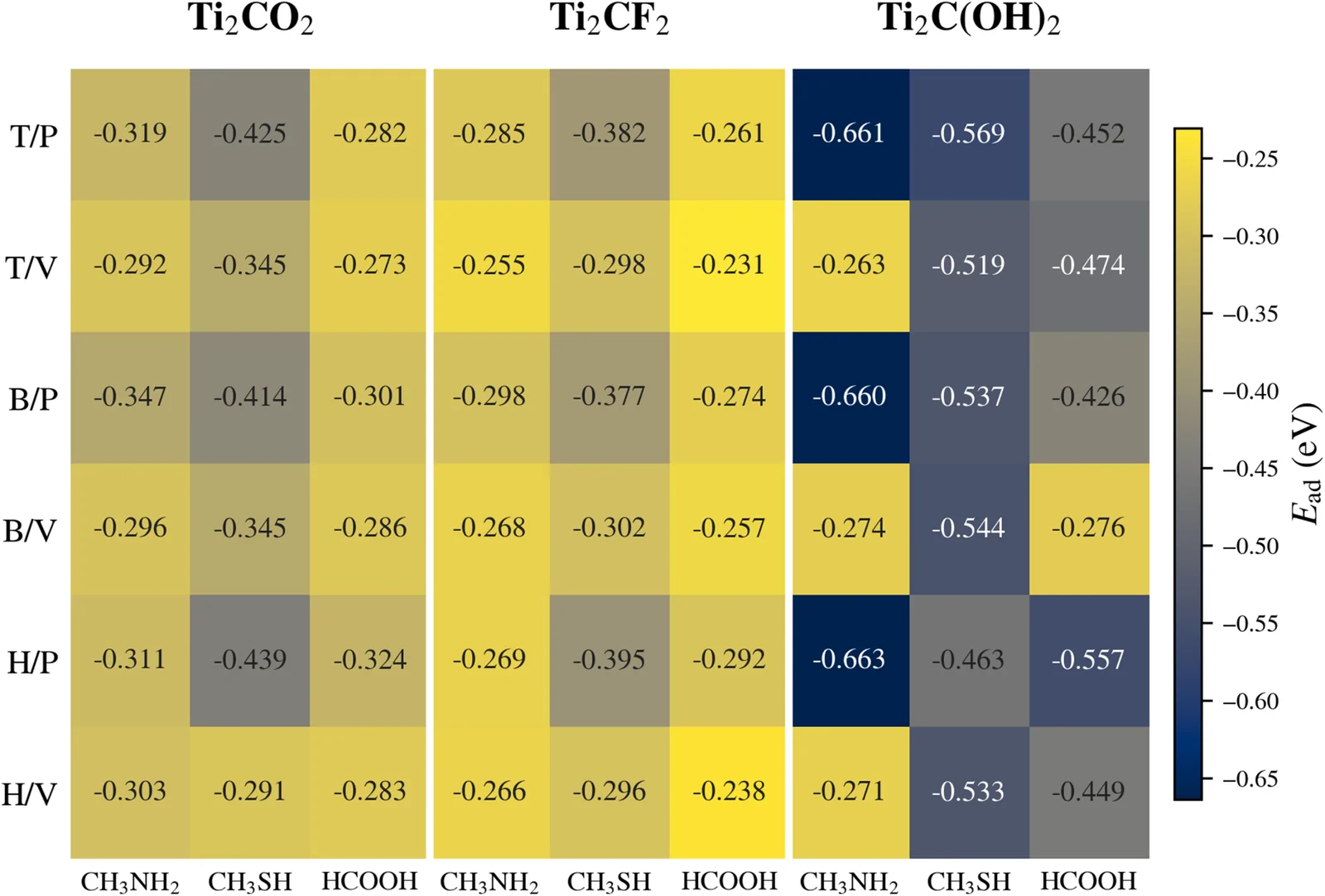
Adsorption energy map for CH_3_NH_2_, CH_3_SH, and HCOOH on Ti_2_CO_2_, Ti_2_CF_2_, and Ti_2_C(OH)_2_ monolayers. Rows correspond to T/P, T/V, B/P, B/V, H/P, and H/V configurations, where T, B, and H denote top, bridge, and hollow sites, and P and V denote parallel and vertical orientations, respectively. Columns are grouped by monolayer and molecule. Each value represents the optimized adsorption energy, *E*_ad_, in eV. Darker blue indicates more negative adsorption energies and stronger adsorption, whereas yellow indicates less negative adsorption energies and weaker adsorption. The F hollow/parallel CH_3_NH_2_ run did not reach the force target and is shown for transparency, not as a converged minimum.

Fig. 4 presents the 18 optimized adsorption configurations on Ti_2_CO_2_, with adsorption energies ranging from −0.439 to −0.273 eV. Among the three molecules, CH_3_SH adsorbs most strongly, followed by CH_3_NH_2_ and HCOOH. The most stable configuration of each molecule adopts a parallel orientation: hollow/parallel for CH_3_SH (−0.439 eV), bridge/parallel for CH_3_NH_2_ (−0.347 eV), and hollow/parallel for HCOOH (−0.324 eV). These results establish moderate adsorption and preferential CH_3_SH binding on Ti_2_CO_2_, providing a reference for assessing how surface termination alters adsorption strength and adsorbate preference.

Fig. 5 presents the 18 optimized adsorption configurations on Ti_2_CF_2_, with adsorption energies ranging from −0.395 to −0.231 eV, indicating the weakest overall adsorption among the three monolayers. As on Ti_2_CO_2_, CH_3_SH adsorbs most strongly, followed by CH_3_NH_2_ and HCOOH. The most stable configuration of each molecule adopts a parallel orientation: hollow/parallel for CH_3_SH (−0.395 eV), bridge/parallel for CH_3_NH_2_ (−0.298 eV), and hollow/parallel for HCOOH (−0.292 eV). Compared with Ti_2_CO_2_, Ti_2_CF_2_ exhibits weaker adsorption while retaining the preference for CH_3_SH.

Fig. 6 presents the 18 optimized adsorption configurations on Ti_2_C(OH)_2_, with adsorption energies ranging from −0.663 to −0.263 eV. In contrast to Ti_2_CO_2_ and Ti_2_CF_2_, which preferentially adsorb CH_3_SH, Ti_2_C(OH)_2_ preferentially adsorbs CH_3_NH_2_ while retaining favorable adsorption of CH_3_SH and HCOOH. The most stable configuration for each molecule adopts a parallel orientation: hollow/parallel for CH_3_NH_2_ (−0.663 eV), top/parallel for CH_3_SH (−0.569 eV), and hollow/parallel for HCOOH (−0.557 eV). The hollow/parallel CH_3_NH_2_/Ti_2_C(OH)_2_ complex exhibits the strongest adsorption among all 54 configurations. Compared with Ti_2_CO_2_ and Ti_2_CF_2_, Ti_2_C(OH)_2_ strengthens the adsorption of all three molecules and shifts adsorbate preference from CH_3_SH to CH_3_NH_2_.

The adsorption energies also reveal the effects of adsorption site and molecular orientation. All nine most stable adsorption complexes adopt a parallel orientation. The largest site-dependent difference in adsorption energy occurs for vertically oriented HCOOH on Ti_2_C(OH)_2_, reaching approximately 0.20 eV between the top site (−0.474 eV) and the bridge site (−0.276 eV). The largest orientation-dependent difference occurs for CH_3_NH_2_ on Ti_2_C(OH)_2_, reaching approximately 0.40 eV between the original parallel configurations (−0.663 to −0.660 eV) and the methyl-down vertical configurations (−0.274 to −0.263 eV). Because CH_3_NH_2_/Ti_2_C(OH)_2_ is also the most strongly bound complex among all 54 configurations, this pronounced orientation dependence demonstrates that molecular alignment strongly influences CH_3_NH_2_ adsorption on Ti_2_C(OH)_2_. Additional nitrogen-facing CH_3_NH_2_ starts can approach the parallel minimum on OH termination; therefore, the large difference observed for the original methyl-down starts should not be generalized to all vertical geometries (SI Section S3).

Taken together, the 54-configuration analysis establishes three principal adsorption trends: adsorption strength follows Ti_2_C(OH)_2_ > Ti_2_CO_2_ > Ti_2_CF_2_; adsorbate preference depends on surface termination, with Ti_2_CO_2_ and Ti_2_CF_2_ preferentially adsorbing CH_3_SH and Ti_2_C(OH)_2_ preferentially adsorbing CH_3_NH_2_; and Ti_2_C(OH)_2_ exhibits the greatest geometry-dependent variations in adsorption energy.

Targeted 3 × 3 supercell calculations were performed for the nine most stable adsorption complexes to determine whether the adsorption strength order and adsorbate preferences obtained with the 2 × 2 supercells were preserved upon increasing the supercell size. As summarized in Table 4, the corresponding adsorption-energy differences ranged from 0.004 to 0.059 eV between the 2 × 2 and 3 × 3 supercells. The 3 × 3 calculations preserved both adsorption trends: adsorption strength followed Ti_2_C(OH)_2_ > Ti_2_CO_2_ > Ti_2_CF_2_ for every molecule, and CH_3_SH remained the preferred adsorbate on Ti_2_CO_2_ and Ti_2_CF_2_, whereas CH_3_NH_2_ remained the preferred adsorbate on Ti_2_C(OH)_2_. These results support the robustness of the adsorption patterns obtained with the 2 × 2 supercells and suggest that, within the supercell sizes examined, interactions between periodically repeated adsorbates do not control the observed qualitative trends. These adsorption-energy checks do not establish coverage convergence of band gaps or work functions.

**Table 4:** Supercell size validation for the most stable adsorption complexes, including adsorption energies in the 2 × 2 (*E*_ad,2×2_) and 3 × 3 (*E*_ad,3×3_) supercells and absolute adsorption energy differences (|Δ*E*|)

Monolayer	Molecule	Most stable configuration	*E*_ad,2×2_ (eV)	*E*_ad,3×3_ (eV)	|Δ*E*| (eV)
Ti_2_CO_2_	CH_3_NH_2_	B/P	−0.347	−0.351	0.004
Ti_2_CO_2_	CH_3_SH	H/P	−0.439	−0.383	0.056
Ti_2_CO_2_	HCOOH	H/P	−0.324	−0.303	0.021
Ti_2_CF_2_	CH_3_NH_2_	B/P	−0.298	−0.288	0.010
Ti_2_CF_2_	CH_3_SH	H/P	−0.395	−0.336	0.059
Ti_2_CF_2_	HCOOH	H/P	−0.292	−0.269	0.023
Ti_2_C(OH)_2_	CH_3_NH_2_	H/P	−0.663	−0.676	0.013
Ti_2_C(OH)_2_	CH_3_SH	T/P	−0.569	−0.539	0.030
Ti_2_C(OH)_2_	HCOOH	H/P	−0.557	−0.548	0.009

### 3.3 Adsorption, recovery, and electronic response

The nine most stable adsorption complexes were examined to compare molecular retention, adsorbate–monolayer proximity, reversibility, and adsorption-induced electronic responses. Table 5 summarizes their adsorption energies, minimum adsorption distances, and estimated molecular residence times. Charge density difference, band structure, and PDOS analyses were used to characterize charge redistribution and adsorbate contributions to the electronic states near the Fermi level. Adsorption-induced work function shifts quantified molecule-dependent changes in the surface potentials of all three monolayers, whereas the corresponding band gap changes provided a complementary quantitative estimate of the model-dependent electronic response of semiconducting Ti_2_CO_2_. Together, these results reveal how the three monolayers differ in molecular retention, reversibility, and electronic response.

**Table 5:** Adsorption energy (*E*_ad_), minimum adsorption distance (*d*_min_), and estimated molecular residence time (*τ*) for the selected adsorption complexes^a^

Monolayer	Molecule	Most stable configuration	*E*_ad_ (eV)	*d*_min_ (Å)	*τ* (s)
Ti_2_CO_2_	CH_3_NH_2_	B/P	−0.347	2.70	6.88 × 10^−7^
Ti_2_CO_2_	CH_3_SH	H/P	−0.439	2.75	2.34 × 10^−5^
Ti_2_CO_2_	HCOOH	H/P	−0.324	3.03	2.74 × 10^−7^
Ti_2_CF_2_	CH_3_NH_2_	B/P	−0.298	2.65	1.03 × 10^−7^
Ti_2_CF_2_	CH_3_SH	H/P	−0.395	2.63	4.37 × 10^−6^
Ti_2_CF_2_	HCOOH	H/P	−0.292	3.02	8.10 × 10^−8^
Ti_2_C(OH)_2_	CH_3_NH_2_	H/P	−0.663	1.75	1.40 × 10^−1^
Ti_2_C(OH)_2_	CH_3_SH	T/P	−0.569	2.22	3.61 × 10^−3^
Ti_2_C(OH)_2_	HCOOH	H/P	−0.557	1.84	2.30 × 10^−3^

a Residence times were estimated by approximating the desorption barrier as the adsorption-energy magnitude (*E*_des_ ≈ |*E*_ad_|). These estimates do not establish macroscopic sensor recovery or capture performance.

#### 3.3.1 Ti_2_CO_2_ adsorption complexes

As summarized in Table 5, CH_3_SH adsorbs most strongly on Ti_2_CO_2_ (−0.439 eV), followed by CH_3_NH_2_ (−0.347 eV) and HCOOH (−0.324 eV). The corresponding minimum adsorption distances are 2.75, 2.70, and 3.03 Å, respectively. The estimated molecular residence times range from 2.74 × 10^−7^ to 2.34 × 10^−5^ s, suggesting rapid desorption within the assumed barrier model and surface regeneration but potentially limiting molecular retention and the duration of an adsorption-induced signal.

As shown in Fig. 8, the Ti_2_CO_2_ adsorption complexes exhibit localized electron accumulation and depletion at the molecule–monolayer interfaces, whereas charge redistribution is minimal beyond the adsorption regions. The band structures show that Ti_2_CO_2_ remains semiconducting after adsorption, with no bands crossing the Fermi level. The corresponding PDOS profiles show that the projected adsorbate states associated with N in CH_3_NH_2_, S in CH_3_SH, and O in HCOOH occur mainly below the Fermi level, with no clear adsorbate-derived states within the band gap or at the Fermi level. Taken together, these results indicate localized charge redistribution without loss of the semiconducting character of Ti_2_CO_2_.

**Fig. 8:**
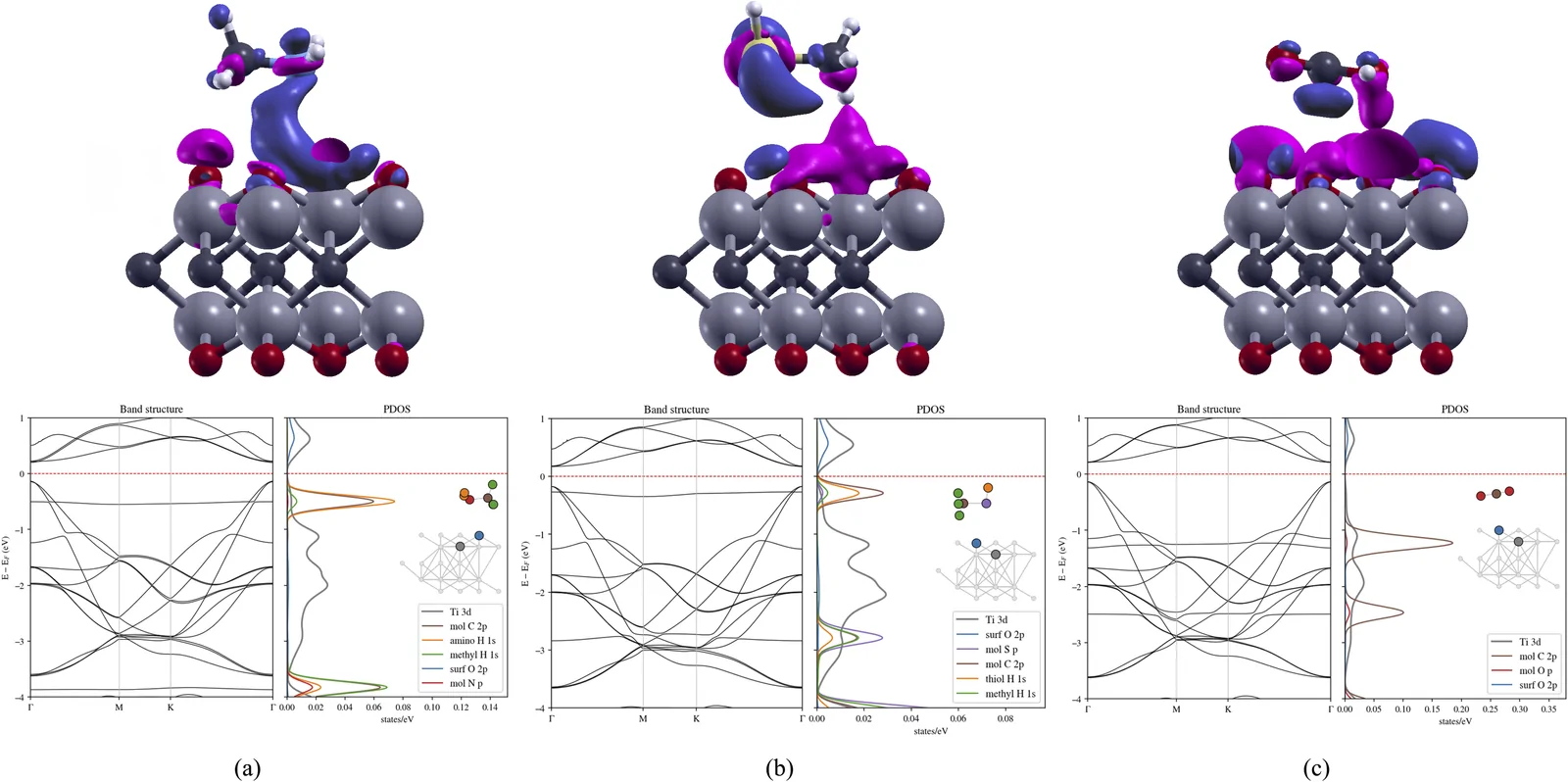
Charge density difference isosurfaces, band structures, and PDOS profiles for the most stable (a) CH_3_NH_2_/Ti_2_CO_2_, (b) CH_3_SH/Ti_2_CO_2_, and (c) HCOOH/Ti_2_CO_2_ adsorption complexes. The top row shows charge density difference isosurfaces, and the bottom row shows the corresponding band structures and PDOS profiles. Magenta and blue indicate electron accumulation and depletion, respectively. The isosurface values are 0.002 e Å^−3^ for CH_3_NH_2_, 0.0015 e Å^−3^ for CH_3_SH, and 0.0012 e Å^−3^ for HCOOH. The Fermi level is set to 0 eV. H, C, N, O, S, and Ti atoms are shown in white, black, blue, red, yellow, and gray, respectively. The atom color inset and PDOS legends identify the atoms and projected orbitals used in the analysis. Different isosurface thresholds prevent quantitative comparison of redistribution magnitudes between panels.

The work functions of the three bare monolayers and their most stable adsorption complexes are summarized in Table 6. The bare work functions follow the order Ti_2_CO_2_ (5.34 eV) > Ti_2_CF_2_ (4.47 eV) > Ti_2_C(OH)_2_ (1.76 eV), demonstrating the strong dependence of work function on termination chemistry. Values follow the extraction and its window diagnostics (SI Table S5).

**Table 6:** Calculated work functions of bare (*Φ*_bare_) and adsorbate-bound (*Φ*_complex_) Ti_2_C monolayers and adsorption-induced work function shifts (Δ*Φ*)^a^

Monolayer	Adsorbate	*Φ*_bare_ (eV)	*Φ*_complex_ (eV)	Δ*Φ* (eV)
Ti_2_CO_2_	CH_3_NH_2_	5.34	4.72	−0.62
Ti_2_CO_2_	CH_3_SH	5.34	5.00	−0.35
Ti_2_CO_2_	HCOOH	5.34	5.47	+0.13
Ti_2_CF_2_	CH_3_NH_2_	4.47	3.93	−0.54
Ti_2_CF_2_	CH_3_SH	4.47	4.25	−0.22
Ti_2_CF_2_	HCOOH	4.47	4.61	+0.14
Ti_2_C(OH)_2_	CH_3_NH_2_	1.76	1.73	−0.04
Ti_2_C(OH)_2_	CH_3_SH	1.76	1.74	−0.03
Ti_2_C(OH)_2_	HCOOH	1.76	1.72	−0.05

a Δ*Φ* values were calculated using the unrounded work function values. Extraction details and potential-window diagnostics are provided in SI Section S5.

For Ti_2_CO_2_, adsorption lowers the work function from 5.34 eV to 4.72 eV for CH_3_NH_2_ and 5.00 eV for CH_3_SH, whereas HCOOH increases it to 5.47 eV, corresponding to shifts of −0.62, −0.35, and +0.13 eV, respectively. The work function shifts therefore vary among the three adsorbates in both magnitude and direction. The absolute magnitude of the work function shift follows CH_3_NH_2_ > CH_3_SH > HCOOH, differing from the adsorption strength order CH_3_SH > CH_3_NH_2_ > HCOOH. HCOOH also produces a work function shift opposite in sign to those of CH_3_NH_2_ and CH_3_SH. This contrast indicates that work function modulation does not follow adsorption strength alone but reflects molecule-dependent charge redistribution and the resulting changes in surface electrostatic potential. Molecular orientation, polarization, charge rearrangement and Fermi-level changes may contribute to the opposite HCOOH sign, but their individual contributions are not resolved here.

Because Ti_2_CO_2_ remains semiconducting after adsorption and no clear adsorbate-derived states appear within the band gap or at the Fermi level, its model-dependent electronic response was also estimated from adsorption-induced band gap changes, as summarized in Table 7. Adsorption widens the band gap from 0.322 eV to 0.335 eV for CH_3_NH_2_ and to approximately 0.343 eV for CH_3_SH and HCOOH, corresponding to increases of 4.10%, 6.44%, and 6.54%, respectively. According to eqn (5) and (6), band gap widening lowers the estimated conductivity, so greater widening produces larger relative changes in estimated conductivity. Therefore, CH_3_SH and HCOOH produce higher model-dependent electronic response estimates of 33.0% and 33.5%, respectively, than the 22.6% estimated for CH_3_NH_2_. Thus, the estimated model-dependent electronic response of Ti_2_CO_2_ arises from adsorption-induced band gap widening rather than the formation of new adsorbate-derived states at the Fermi level. Previous first-principles work has likewise demonstrated the sensing potential of Ti_2_CO_2_ for DNA/RNA nucleobases.^74^ Although CH_3_SH and HCOOH produce nearly identical band-gap-based responses of 33.0% and 33.5%, they induce opposite-sign work function shifts of −0.35 and +0.13 eV, respectively. These complementary electronic signatures could therefore support differentiation between the two molecules and motivate further testing of Ti_2_CO_2_ for semiconductor sensing.

**Table 7:** Band gap before (*E*_g,1_) and after (*E*_g,2_) adsorption, percentage band gap change (Δ*E*_g_), and estimated sensing response (*S*) for the most stable Ti_2_CO_2_ adsorption complexes^a^

Adsorption complex	*E*_g,1_ (eV)	*E*_g,2_ (eV)	Δ*E*_g_ (%)	*S* (%)
CH_3_NH_2_/Ti_2_CO_2_	0.322	0.335	+4.10	22.6
CH_3_SH/Ti_2_CO_2_	0.322	0.343	+6.44	33.0
HCOOH/Ti_2_CO_2_	0.322	0.343	+6.54	33.5

a Δ*E*_g_ and *S* were calculated using the unrounded band gaps.

For CH_3_SH/Ti_2_CO_2_, denser *k*-point sampling and reduced occupation smearing preserve the positive adsorption-induced band-gap change. These representative checks support the observed trend, although full relaxation and coverage convergence remain to be established (SI Section S4).

#### 3.3.2 Ti_2_CF_2_ adsorption complexes

Compared with Ti_2_CO_2_, Ti_2_CF_2_ adsorbs all three molecules more weakly, with adsorption energies of −0.298 eV for CH_3_NH_2_, −0.395 eV for CH_3_SH, and −0.292 eV for HCOOH. The corresponding minimum adsorption distances are 2.65, 2.63, and 3.02 Å, respectively. The estimated molecular residence times range from 8.10 × 10^−8^ to 4.37 × 10^−6^ s, indicating faster desorption within the assumed barrier model than on Ti_2_CO_2_ but weaker molecular retention.

As shown in Fig. 9, the Ti_2_CF_2_ adsorption complexes exhibit electron accumulation and depletion localized near the molecule–monolayer contact regions. The band structures show that Ti_2_CF_2_ retains its metallic character after adsorption. The corresponding PDOS profiles show that the projected states associated with N in CH_3_NH_2_, S in CH_3_SH, and O in HCOOH occur mainly below the Fermi level, with only minor adsorbate contributions near the Fermi level. Collectively, these results indicate that adsorption produces spatially confined charge redistribution, while the near-Fermi electronic structure remains dominated by Ti_2_CF_2_ states, with only minor adsorbate contributions.

**Fig. 9:**
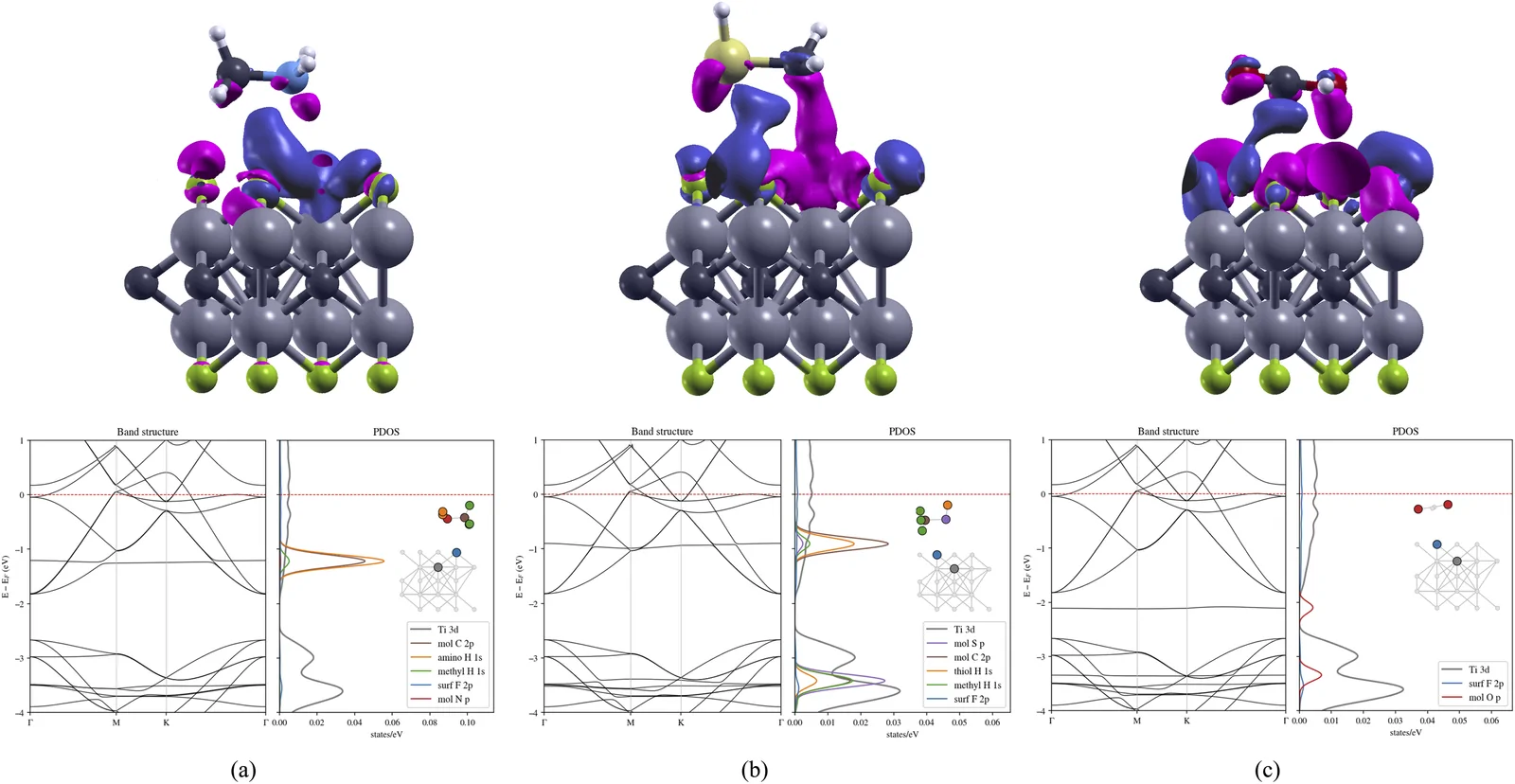
Charge density difference isosurfaces, band structures, and PDOS profiles for the most stable adsorption complexes of (a) CH_3_NH_2_, (b) CH_3_SH, and (c) HCOOH on Ti_2_CF_2_. The top and bottom rows show charge density difference isosurfaces and the corresponding band structures with PDOS, respectively. Magenta and blue regions indicate electron accumulation and depletion, respectively. The isosurface values are 0.002 e Å^−3^ for CH_3_NH_2_, 0.0009 e Å^−3^ for CH_3_SH, and 0.0008 e Å^−3^ for HCOOH. The Fermi level is set to 0 eV. H, C, N, O, S, F, and Ti atoms are shown in white, black, blue, red, yellow, green, and gray, respectively. The atom color inset and PDOS legends identify the atoms and projected orbitals used in the analysis. Different isosurface thresholds prevent quantitative comparison of redistribution magnitudes between panels.

Bare Ti_2_CF_2_ has a work function of 4.47 eV. Adsorption changes the work function to 3.93 eV for CH_3_NH_2_, 4.25 eV for CH_3_SH, and 4.61 eV for HCOOH, corresponding to shifts of −0.54, −0.22, and +0.14 eV, respectively. The work function response therefore differs in both magnitude and direction among the three adsorbates, with the largest decrease for CH_3_NH_2_, a smaller decrease for CH_3_SH, and an increase for HCOOH. Despite comparatively weak adsorption and only minor adsorbate contributions near the Fermi level, these distinct shifts indicate that work function modulation does not directly track either adsorption strength or near-Fermi adsorbate participation. Thus, among the three monolayers, Ti_2_CF_2_ exhibits the shortest estimated molecular residence times and weakest adsorption, suggesting weaker molecular retention under the studied conditions. However, its molecule-dependent work function shifts of −0.54 to +0.14 eV support its potential for work-function-based sensing.

#### 3.3.3 Ti_2_C(OH)_2_ adsorption complexes

In contrast to Ti_2_CO_2_ and Ti_2_CF_2_, Ti_2_C(OH)_2_ exhibits the strongest adsorption for each molecule. As summarized in Table 5, CH_3_NH_2_ adsorbs most strongly, with an adsorption energy of −0.663 eV, the shortest adsorption distance of 1.75 Å, and an estimated molecular residence time of 1.40 × 10^−1^ s. CH_3_SH and HCOOH also adsorb favorably, with adsorption energies of −0.569 and −0.557 eV, adsorption distances of 2.22 and 1.84 Å, and estimated molecular residence times of 3.61 × 10^−3^ and 2.30 × 10^−3^ s, respectively. Compared with the corresponding complexes on Ti_2_CO_2_ and Ti_2_CF_2_, Ti_2_C(OH)_2_ combines stronger adsorption and shorter adsorption distances with subsecond estimated molecular residence times, indicating stronger binding with preliminary desorption trends. These results identify Ti_2_C(OH)_2_ as the strongest-binding monolayer, particularly for CH_3_NH_2_; useful reversible capture is not established by these estimates alone.

The shortest OH/CH_3_NH_2_ contact is a nearly linear surface O–H⋯N hydrogen bond (H⋯N ≈ 1.75 Å, O⋯N ≈ 2.76 Å, angle ≈ 178°), distinct from the N–Ti binding.^39^ O/F surfaces lack this hydroxyl donor. Their CH_3_SH preference is established energetically, but the available results do not isolate a dominant dispersion or orbital contribution. Contact geometries and representative quantitative charge-redistribution profiles are provided in SI Section S7.

The charge density difference plots reveal adsorption-induced interfacial charge redistribution in the Ti_2_C(OH)_2_ complexes. As shown in Fig. 10, all three complexes exhibit clear electron accumulation and depletion localized at the molecule–monolayer interfaces. For CH_3_NH_2_/Ti_2_C(OH)_2_, the redistribution is concentrated near the shortest molecule–monolayer contact, consistent with its strongest adsorption and shortest adsorption distance, while the CH_3_SH and HCOOH complexes also show clear interfacial redistribution.

**Fig. 10:**
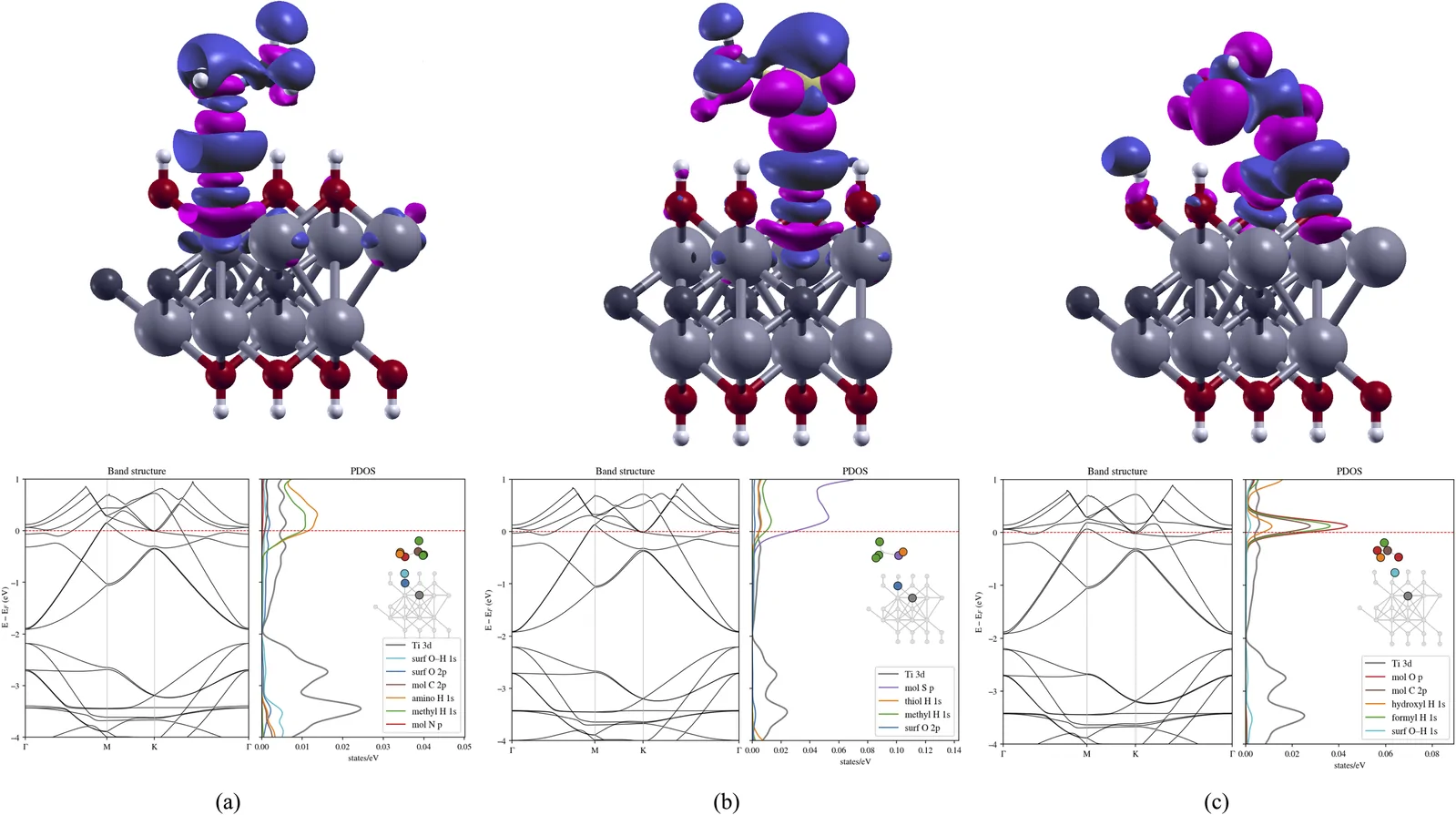
Charge density difference isosurfaces, band structures, and PDOS profiles for the most stable adsorption complexes of (a) CH_3_NH_2_, (b) CH_3_SH, and (c) HCOOH on Ti_2_C(OH)_2_. The top and bottom rows show charge density difference isosurfaces and the corresponding band structures with PDOS, respectively. Magenta and blue regions indicate electron accumulation and depletion, respectively. The isosurface values are 0.004 e Å^−3^ for CH_3_NH_2_, 0.003 e Å^−3^ for CH_3_SH, and 0.005 e Å^−3^ for HCOOH. The Fermi level is set to 0 eV. H, C, N, O, S, and Ti atoms are shown in white, black, blue, red, yellow, and gray, respectively. The atom color inset and PDOS legends identify the atoms and projected orbitals used in the analysis. Different isosurface thresholds prevent quantitative comparison of redistribution magnitudes between panels.

The band structures in Fig. 10 show that Ti_2_C(OH)_2_ retains metallic character after adsorption. The corresponding PDOS profiles reveal molecule-dependent differences in the adsorbate contributions to the electronic structure near the Fermi level. For CH_3_NH_2_/Ti_2_C(OH)_2_, the dominant N 2p peak lies well below the Fermi level, with no prominent N-derived peak directly at the Fermi level. Thus, the strongest adsorption observed is associated with charge redistribution and occupied N-derived states below the Fermi level rather than a prominent N-derived contribution at the Fermi level.

By comparison, the S 3p peak for CH_3_SH/Ti_2_C(OH)_2_ and the O 2p peak for HCOOH/Ti_2_C(OH)_2_ lie closer to the Fermi level and show greater energy overlap with Ti_2_C(OH)_2_-derived states than the N 2p peak for CH_3_NH_2_/Ti_2_C(OH)_2_. Combined with the charge redistribution, these overlapping states are consistent with greater adsorbate participation in the near-Fermi electronic structure for CH_3_SH and HCOOH than for CH_3_NH_2_. Adsorption energy and PDOS therefore characterize complementary aspects of the molecule–monolayer interaction: adsorption energy quantifies interaction strength, whereas PDOS identifies adsorbate contributions to electronic states near the Fermi level. Consequently, the molecule retained most strongly need not produce the greatest adsorbate contribution near the Fermi level, highlighting an important distinction between molecular capture and sensing-relevant electronic response.

Bare Ti_2_C(OH)_2_ has a calculated work function of 1.76 eV, the lowest among the three monolayers and consistent with the ultralow work functions reported for OH-terminated MXenes.^40^ Adsorption decreases the work function to 1.73 eV for CH_3_NH_2_, 1.74 eV for CH_3_SH, and 1.72 eV for HCOOH, corresponding to small shifts of −0.04, −0.03, and −0.05 eV, respectively. In contrast to Ti_2_CO_2_ and Ti_2_CF_2_, which exhibit larger molecule-dependent shifts with both negative and positive values, Ti_2_C(OH)_2_ produces only small negative shifts for all three adsorbates. These small shifts do not establish molecular differentiation through work function alone. The similar shifts, despite differences in adsorption strength and near-Fermi adsorbate contributions, indicate that work-function response, adsorption strength, and PDOS provide complementary measures of the molecule–monolayer interaction. Accordingly, Ti_2_C(OH)_2_ remains the strongest-binding monolayer among the three monolayers, particularly for CH_3_NH_2_, but the present work function results do not establish whether the adsorbates can be distinguished. The small shifts are comparable to the 0.044–0.059 eV potential spans in the complex averaging windows, so their fine ordering and sign stability require further convergence checks (SI Table S5).

### 3.4 Implications and limitations

Taken together, the work function results show that both the magnitude and direction of the shifts vary with surface termination and adsorbate identity and do not directly track adsorption strength. Ti_2_CO_2_ and Ti_2_CF_2_ exhibit pronounced molecule-dependent responses: CH_3_NH_2_ and CH_3_SH decrease the work function, whereas HCOOH increases it. For Ti_2_C(OH)_2_, the present extraction gives small negative shifts for all three adsorbates; their sign and fine ordering require further validation. Across the three adsorbates, the work function shifts cover ranges of 0.75 eV for Ti_2_CO_2_ and 0.68 eV for Ti_2_CF_2_, but only 0.02 eV for Ti_2_C(OH)_2_. This divergence suggests that work function modulation reflects adsorbate-dependent charge redistribution and the associated changes in surface electrostatic potential rather than adsorption strength alone. Ti_2_C(OH)_2_ therefore exhibits the strongest adsorption and the smallest extracted work-function shifts, but these shifts do not establish molecular differentiation or a ranking of sensing performance.

First-principles studies have reported estimated recovery times spanning submicrosecond to subsecond timescales.^75,76^ Here the estimates of 2.30 × 10^−3^ to 1.40 × 10^−1^ s for Ti_2_C(OH)_2_ and 8.10 × 10^−8^ to 2.34 × 10^−5^ s for Ti_2_CO_2_/Ti_2_CF_2_ should be interpreted as molecular residence-time proxies and preliminary desorption trends. The adsorption-energy magnitude approximates the desorption barrier; these are not independently calculated barriers or macroscopic sensor-recovery times.^66^ Sensitivity to attempt frequency, temperature and barrier assumptions is quantified in SI Table S6. Actual regeneration can also depend on humidity, defects, competing species, readsorption, diffusion and device transport. Without adsorption free energies or equilibrium coverages at specified temperature and pressure, these estimates do not establish useful reversible capture.

Uniformly terminated models isolate termination effects that are difficult to separate in experimentally synthesized mixed-termination MXenes. Schultz *et al.* showed that the work functions of mixed-termination Ti_3_C_2_T_*x*_ surfaces cannot be reproduced by simple averages of uniformly terminated models because local dipole arrangements also contribute.^33^ The present comparison therefore establishes distinct adsorption and electronic-response profiles for O-, F-, and OH-terminated Ti_2_C monolayers, rather than predicting the behavior of a specific mixed-termination Ti_2_CT_*x*_ surface. Mixed terminations also introduce local adsorption and hydrogen-bond environments absent from the uniform models; their responses need not lie between the pure-termination results.

PBE-based adsorption energies and electronic properties depend on the exchange–correlation and dispersion approximations. Semilocal gaps and their derived response estimates may differ from experiment, and numerical convergence does not remove functional error. Vibrational free energies, solvent, oxidation, finite-temperature surface changes and device transport are not represented here.

The metallic character of Ti_2_CF_2_ and Ti_2_C(OH)_2_ does not preclude either sensing or adsorption-based capture. Schedin *et al.* showed that adsorption on graphene can produce resistance changes through shifts in carrier concentration, demonstrating that adsorption-induced electrical responses can arise without band gap modulation.^77^ Koh *et al.* showed that gas-induced interlayer swelling contributes to sensing in Ti_3_C_2_T_*x*_ films, highlighting interlayer effects not represented in isolated monolayer models.^78^ Experimental Ti_3_C_2_T_*x*_ studies have also demonstrated uptake of diverse species, supporting the broader feasibility of conductive titanium carbide MXenes as capture materials.^25,47,49^ Accordingly, the calculated work function shifts, charge redistribution, and electronic structure changes near the Fermi level provide sensing-relevant indicators, while the adsorption energies and estimated molecular residence times provide relative binding and preliminary desorption trends. Kim *et al.* demonstrated adsorption-induced electrical responses to acetone and ethanol in metallic Ti_3_C_2_T_*x*_ sensors.^41^ Together with the interlayer-swelling results,^78^ this provides experimental context; differing compositions, mixed terminations and film morphologies prevent direct validation of the present Ti_2_C response magnitudes. The present novelty is the controlled comparison of three termination chemistries and three molecular motifs on the same Ti_2_C backbone.

These computational indicators do not alone establish practical sensing or capture performance. Future work should test whether the predicted trends persist on mixed-termination surfaces,^34^ in solvated environments,^79^ and under competitive adsorption conditions.^80^ Such validation should combine explicit calculations of desorption barriers and charge transport with sensing and capture experiments.

## 4. Conclusion

Dispersion-corrected first-principles DFT calculations revealed how O, F, and OH terminations shape adsorption strength, preliminary desorption trends, and electronic response for CH_3_NH_2_, CH_3_SH, and HCOOH on Ti_2_C monolayers. Ti_2_CO_2_ retained its semiconducting character and exhibited complementary model-dependent band-gap and molecule-dependent work-function responses, making it the most promising semiconductor sensing candidate among the three monolayers. Ti_2_C(OH)_2_ exhibited the strongest adsorption, particularly for CH_3_NH_2_; its short molecular residence-time estimates suggest preliminary desorption trends but do not establish reversible capture performance. Its small and similar work-function shifts indicate that work function alone provides limited molecular differentiation. Ti_2_CF_2_ exhibited the weakest adsorption and shortest estimated molecular residence times, yet its distinct molecule-dependent work-function response supports its potential for further investigation of work-function-based sensing.

These findings demonstrate that the termination producing the strongest adsorption does not necessarily yield the most distinctive electronic response. Accordingly, adsorption strength, preliminary desorption trends, near-Fermi electronic structure, band gap modulation, and work function shifts capture distinct yet complementary aspects of molecule–MXene interactions rather than defining a single performance hierarchy. This study extends previous experimental and computational MXene research by linking O-, F-, and OH-termination effects on the same Ti_2_C backbone to functional-group-dependent adsorption, preliminary desorption trends, and distinct electronic-response profiles. More broadly, this controlled comparison reveals how termination chemistry produces distinct adsorption and electronic-response profiles relevant to further studies of semiconductor sensing, work-function-based sensing, and reversible capture. The response estimates remain conditional on the assumed conductivity model, and the available tests do not establish full force or coverage convergence.

## Conflicts of interest

There are no conflicts to declare.

## Supplementary Material

RA-OLF-D6RA07364F-s001

RA-OLF-D6RA07364F-s002

## Data Availability

The complete set of ABINIT input and output files and relaxed structures underlying this study is openly available on Zenodo at https://doi.org/10.5281/zenodo.21817213. Supplementary information (SI): optimized atomic structures of the isolated molecules, the bare Ti_2_CO_2_, Ti_2_CF_2_, and Ti_2_C(OH)_2_ monolayers, and the nine most stable adsorption complexes. See DOI: https://doi.org/10.1039/d6ra07364f.
