# Supplementary material for: Sensing and reversible capture of methylamine, methanethiol, and formic acid on Ti2C-based MXene monolayers: a DFT study
Source: RSC Adv. 2026 Nov 2. Online ahead of print. doi: 10.1039/d6ra07364f (PMC13725747; doi:10.1039/d6ra07364f)
Supplement: Supplementary file 1 [file RA-OLF-D6RA07364F-s002.pdf]

Supplementary Information (SI) for RSC Advances.
This journal is © The Royal Society of Chemistry

Supplementary Information

Sensing and reversible capture of methylamine, methanethiol, and formic acid on Ti₂C-based MXene monolayers: a DFT study

S1. Structures and computational parameters

The companion all_structures.xyz contains 15 frames: three isolated molecules, three bare monolayers and nine selected adsorption complexes. It is a selected-structure set, not all screened configurations. Geometric cell lengths and vacuum gaps below are calculated from these coordinates. A vacuum gap is the cell height minus the full z extent of the atoms, not the total cell height itself.

Termination	ecut / pawecutdg (Ha)	Production 2 × 2 mesh	Smearing (Ha)	Relaxation c (Å)
O	38 / 76	6 × 6 × 1	0.002	20
F	40 / 80	6 × 6 × 1	0.002	20
OH	35 / 70	6 × 6 × 1	0.002	20

Table S1a. Production adsorption parameters. The production targets are 1 × 10⁻⁸ Ha for electronic energy and 5 × 10⁻⁴ Ha/bohr for force, with PBE and D3(BJ). The common primitive in-plane constant is 3.02999 Å. Exceptions are specified below. Bare O/F work-function references use 20 Å cells, bare OH uses 30 Å, and all selected 2 × 2 work-function complexes use 30 Å. The representative 3 × 3 work-function pair uses 20 Å cells.

Structure	Cell height (Å)	Atomic z extent (Å)	Periodic gap (Å)
slab_Ti2CO2	20.000	4.413	15.587
slab_Ti2CF2	20.000	4.796	15.204
slab_Ti2COH2	20.000	6.794	13.206
Ti2CO2_CH3NH2	20.000	8.398	11.602
Ti2CO2_CH3SH	20.000	8.797	11.203
Ti2CO2_HCOOH	20.000	7.454	12.546
Ti2CF2_CH3NH2	20.000	8.717	11.283
Ti2CF2_CH3SH	20.000	9.131	10.869
Ti2CF2_HCOOH	20.000	7.779	12.221
Ti2COH2_CH3NH2	20.000	10.165	9.835
Ti2COH2_CH3SH	20.000	10.370	9.630

Structure	Cell height (Å)	Atomic z extent (Å)	Periodic gap (Å)
Ti2COH2_HCOOH	20.000	9.549	10.451

Table S1b. Geometric gaps in the supplied selected production structures. These are not work-function single-point cell dimensions and do not establish electrostatic convergence. PAW settings must be reproduced with the actual datasets or generation inputs used; the element configurations and radii are listed in main-text Table 1.

S2. Common-cell strain sensitivity

System	ΔE_{ad} (meV)	Force qualification
O / CH₃SH	−4.9	Slab and complex meet production target
F / CH₃SH	−0.2	Slab and complex meet production target
OH / CH₃NH₂	+8.59	Bare slab uses looser tolerance

Table S2. E_{ad}(own lattice) – E_{ad}(common lattice) for three representative pairs, calculated from the supplied raw energies. The OH bare slab used tolmxf = 2 × 10⁻^{3} Ha/bohr and achieved 1.5385 × 10⁻^{3} Ha/bohr; the OH complex met the production target. The OH result is indicative, not a fully matched-tolerance validation. These tests address selected adsorption energies and do not establish unchanged electronic properties or every configurational ranking.

S3. Extended methylamine orientation search

Energies are relative to the lowest converged parallel configuration for each termination. N-down and methyl-down identify starting orientations; relaxation can change the final orientation. All force magnitudes and targets are in Ha/bohr. Unconverged energies are retained only for transparency and excluded from converged-minimum selection.

Surface	Site	Start	ΔE (eV)	Max force	Converged
O	hollow	parallel	+0.037	0.000394	Yes
O	hollow	N down	+0.063	0.000486	Yes
O	hollow	methyl down	+0.044	0.000412	Yes
O	bridge	parallel	+0.000	0.000419	Yes
O	bridge	N down	+0.038	0.000486	Yes
O	bridge	methyl down	+0.052	0.000287	Yes
O	top	parallel	+0.028	0.00045	Yes
O	top	N down	+0.071	0.000414	Yes
O	top	methyl down	+0.056	0.000353	Yes
F	hollow	parallel	+0.029	0.00158	No

Surface	Site	Start	ΔE (eV)	Max force	Converged
F	hollow	N down	+0.047	0.000456	Yes
F	hollow	methyl down	+0.032	0.000474	Yes
F	bridge	parallel	+0.000	0.000435	Yes
F	bridge	N down	+0.031	0.00037	Yes
F	bridge	methyl down	+0.030	0.000388	Yes
F	top	parallel	+0.013	0.000412	Yes
F	top	N down	+0.056	0.000468	Yes
F	top	methyl down	+0.043	0.000439	Yes
OH	hollow	parallel	+0.000	0.000442	Yes
OH	hollow	N down	+0.007	0.000399	Yes
OH	hollow	methyl down	+0.393	0.000492	Yes
OH	bridge	parallel	+0.004	0.000417	Yes
OH	bridge	N down	+0.007	0.00286	No
OH	bridge	methyl down	+0.390	0.000307	Yes
OH	top	parallel	+0.002	0.000482	Yes
OH	top	N down	+0.039	0.000365	Yes
OH	top	methyl down	+0.401	0.000482	Yes

Table S3. Extended 27-start CH₃NH₂ set. Target: 5 × 10⁻⁴ Ha/bohr. The OH bridge/N-down and F hollow/parallel runs are unconverged. The OH hollow/N-down result is approximately 6.8 meV above the converged parallel minimum; the best methyl-down state is approximately 0.390 eV higher. The O/F ≤7 meV comparison refers to best-of-orientation energies, not every same-site comparison.

S4. Representative electronic-property checks

Test	Bare Eg (eV)	Complex Eg (eV)	ΔEg / interpretation
Production (reported)	≈0.3222	≈0.3429	≈+20.7 meV; rounded baseline
A: 12 × 12 × 1	0.32256	0.34363	+21.06 meV from unrounded output
B: smearing 0.001 Ha	0.32071	0.34246	+21.74 meV from unrounded output
C: additional relaxation	No matched tighter reference	0.34142	Not tighter-force convergence

Table S4a. CH₃SH/Ti₂CO₂ sensitivity tests. The production row is the reported rounded baseline; it is not an independently reconstructed unrounded result. Differences in A/B use unrounded eigenvalues and may differ by 0.01 meV from subtracting displayed gaps. C stopped at 6.07 × 10⁻⁴ Ha/bohr, exceeding the 1 × 10⁻⁴ target and 5 × 10⁻⁴ production criterion. The original bare reference does not turn C into a matched tighter-relaxation validation.

3 × 3 window check	Φbare (eV)	Φcomplex (eV)	ΔΦ (eV)
Centre ±2 Å	5.3291	5.0092	−0.3199
Centre ±1 Å	—	—	≈−0.3013
±1 Å, centre shifted +1 Å	—	—	≈−0.2621

Table S4b. Work-function extraction sensitivity for the existing 3 × 3 bare/complex pair. The baseline complex window spans 0.349 eV. The same centre-offset adjustment is applied to the bare reference for the shifted test. These are sensitivity checks, not choices for recovering a preferred value. The negative shift persists across these windows; precise magnitude and a 26 meV coverage difference are not established. The 3 × 3 calculations use 20 Å cells, while the 2 × 2 complex uses 30 Å. Consequently, coverage and cell-height effects are mixed.

S5. Work-function extraction diagnostics

The vacuum level is the mean planar potential in a periodic 4 Å-wide window centred half a cell away from the mean atomic z coordinate. Window endpoints can exceed the cell length because periodic wrapping is used. The final applicable Fermi level is subtracted. No flat plateau, side-specific asymptotic vacuum level or dipole-correction convergence is claimed. Values below reproduce the latest extraction summary; window standard deviations and spans measure variation, not statistical uncertainty.

System	Φ (eV)	ΔΦ (eV)	Window (Å)	Std (meV)	Span (meV)
Ti_2CO_2	5.3419	—	14.022–18.022	12.36	69.32

System	Φ (eV)	ΔΦ (eV)	Window (Å)	Std (meV)	Span (meV)
Ti2CF2	4.4724	—	14.022–18.022	9.94	48.14
Ti2COH2	1.7647	—	28.000–32.000	12.15	60.73
Ti2CO2_CH3NH2	4.7227	-0.6192	27.359–31.359	69.86	242.62
Ti2CO2_CH3SH	4.9966	-0.3453	27.085–31.085	38.48	131.83
Ti2CO2_HCOOH	5.4691	+0.1272	27.480–31.480	12.55	51.45
Ti2CF2_CH3NH2	3.9284	-0.5440	27.437–31.437	59.98	220.49
Ti2CF2_CH3SH	4.2491	-0.2233	27.130–31.130	25.34	104.00
Ti2CF2_HCOOH	4.6119	+0.1395	27.539–31.539	14.33	63.83
Ti2COH2_CH3NH2	1.7271	-0.0377	27.435–31.435	9.30	43.72
Ti2COH2_CH3SH	1.7364	-0.0283	27.254–31.254	11.33	59.40
Ti2COH2_HCOOH	1.7189	-0.0458	27.427–31.427	12.60	51.99

Table S5. Extraction diagnostics for Table 6. Small OH shifts require particular caution because their size is comparable to the window variation.

S6. Residence-time sensitivity

The assumed model is τ = ν⁻^{1} exp(E_{des}/kBT), with E_{des} ≈ |E_{ad}|. Changing ν rescales τ inversely. This is not a new transition-state or free-energy calculation. For OH/CH₃NH₂ with |E_{ad}| = 0.663 eV:

T (K)	τ at 10^{11} s⁻^{1} (s)	τ at 10^{12} s⁻^{1} (s)	τ at 10^{13} s⁻^{1} (s)
275	14.2	1.42	0.142
300	1.38	0.138	0.0138
325	0.191	0.0191	0.00191

Table S6. Sensitivity to temperature and attempt frequency. Values use the displayed 0.663 eV energy; small differences from main-text values reflect rounding of the original adsorption energies. At 300 K, an assumed barrier increase/decrease of 0.05 eV multiplies/divides τ by about 6.9. These are neither macroscopic sensor-recovery times nor evidence of equilibrium capture capacity.

S7. Contact geometry and exported-density redistribution

Complex	Shortest contact / D–H···A	Distance (Å)	D–H···A angle (°)
Ti2CO2_CH3NH2	C–H···O (molecular donor)	2.702	99.4
Ti2CO2_CH3SH	C–H···O (molecular donor)	2.752	113.1
Ti2CO2_HCOOH	C–H···O (molecular donor)	3.035	104.1
Ti2CF2_CH3NH2	C–H···F (molecular donor)	2.649	100.4

Complex	Shortest contact / D–H···A	Distance (Å)	D–H···A angle (°)
Ti2CF2_CH3SH	C–H···F (molecular donor)	2.630	113.6
Ti2CF2_HCOOH	C–H···F (molecular donor)	2.974	104.7
Ti2COH2_CH3NH2	O–H···N (surface donor)	1.750	177.7
Ti2COH2_CH3SH	O–H···S (surface donor)	2.217	170.9
Ti2COH2_HCOOH	O–H···O (surface donor)	1.841	161.7

Table S7a. Geometries computed from all_structures.xyz with in-plane periodic images. An angle is shown only when the shortest contact includes H bonded to a same-fragment heavy atom within 1.55 Å; this geometric rule does not classify every contact as a hydrogen bond. The OH/CH₃NH₂ contact is short and nearly linear; the O/F contacts alone do not establish dispersion dominance.

For the representative charge analysis, $\bar{n}(z)$ is the planar-averaged electron number density. We define $Δ\bar{n}(z) = \bar{n}_{complex}(z) - \bar{n}_{slab}(z) - \bar{n}_{mol}(z)$, $λ(z) = AΔ\bar{n}(z)$, and $Q(z) = □_{0}^{z} □(z')dz'$, where A is the in-plane cell area and the integral starts at the cell origin. All calculations use the same cell and grid, with fragment geometries fixed to the adsorption complex. Q_{mol} integrates the electron-number difference above the geometric interface plane; positive values indicate electron accumulation. These descriptors use exported ABINIT PAW density data and depend on the density representation and region boundary; they are not Bader charge transfers or physical dipole moments.

Complex	Peak |Q| (e)	Q_{mol} (e)	Dividing z (Å)
O / CH₃SH	0.0209	−0.0201	9.216
F / CH₃SH	0.00664	−0.00176	9.365
OH / CH₃NH₂	0.0653	+0.0594	10.411

Table S7b. Quantities from the supplied planar profiles. The total integral cancels to approximately 10⁻⁸ e. These representative complexes were selected to compare the strongest-binding case on each termination. Because the adsorbate differs for OH, this is not a fixed-molecule termination ranking. Peak OH rearrangement is approximately 3× O and 10× F. Neither small transfer nor peak rearrangement alone establishes physisorption.

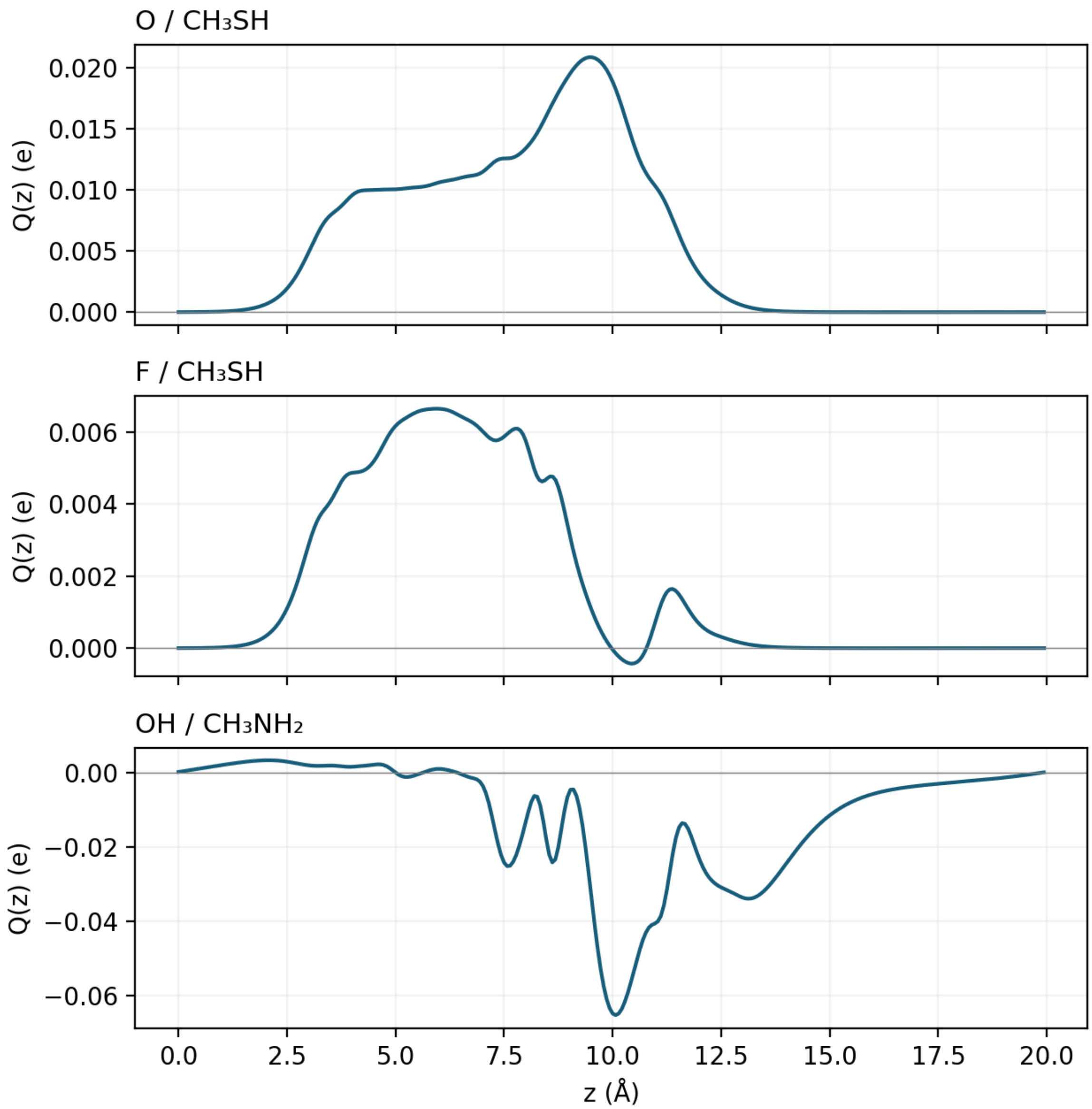

Figure S1. Cumulative exported-density redistribution Q(z) for the three representative complexes. Curves share the same definition, but note the different vertical-axis ranges. Peak magnitudes are given in Table S7b.
